# From review to synthesis: A step-by-step methodological guide to systematic reviews and multilevel meta-analyses

**DOI:** 10.3758/s13428-026-02961-x

**Published:** 2026-06-16

**Authors:** Meilan Hu, Paye Shin Koh, Xun Ci Soh, Andree Hartanto, Nadyanna M. Majeed

**Affiliations:** 1 School of Social Sciences, Singapore Management University, 10 Canning Rise, Singapore, 179873 Singapore; 2 Faculty of Arts and Social Sciences, National University of Singapore, Singapore, Singapore

**Keywords:** Systematic review, Meta-analysis, Multilevel analysis, Tutorial

## Abstract

We provide a step-by-step guide on conducting a quantitative systematic review (i.e., meta-analysis) using the open-source programming language *R*, as well as conducting a multilevel meta-analysis, in contexts where effect sizes are non-independent (e.g., multiple studies from the same lab). Quantitative systematic reviews offer researchers a method for synthesizing large bodies of literature, helping clarify inconsistent findings, identify research gaps, and refine theoretical models. However, existing tutorials often assume prior knowledge and/or experience, often overlooking foundational concepts. To address this gap, a comprehensive walkthrough of the systematic review process is presented, covering pre-registration, literature search and retrieval, screening, risk of bias assessment, and data extraction following the PRISMA framework. We then present detailed guidance on how to conduct both traditional and multilevel meta-analyses in *R*. Specifically, the tutorial explains how to estimate overall meta-analytic effect sizes when effect sizes are independent (traditional meta-analysis) and when effect sizes are nested within labs (multilevel meta-analysis). Procedures for assessing heterogeneity, testing for publication bias, and conducting moderation analyses are also covered. To accompany this tutorial, we supplement annotated *R* scripts and *R* notebooks to support transparency, reproducibility, and accessibility for researchers of all levels of experience.

**Supplementary Information:**

The online version contains supplementary material available at 10.3758/s13428-026-02961-x.

## Introduction

Quantitative systematic reviews offer significant benefits for synthesizing research findings (Kepes et al., 2013; Lee, 2018; Lima et al., 2022; Trikalinos et al., 2008), contributing to a growing number of instructional tutorials designed to guide researchers through the systematic review and meta-analysis processes (Hansen et al., 2022; Lima et al., 2022; Siddaway et al., 2019; Yannascoli et al., 2013). However, many of these tutorials may presume prior familiarity with technical software and research procedures and may omit foundational guidance in research methodology and statistical programming. The current tutorial addresses this gap by offering a beginner-friendly option that guides researchers through the entire process of conducting a quantitative systematic review, from pre-registration to analysis, while ensuring simplicity, accessibility, and research transparency. Moreover, limited guidance exists for situations in which the traditional assumption of independent effect sizes (i.e., unrelated sources) is violated, which can be addressed with a multilevel meta-analytic approach. Therefore, the objectives of this tutorial are threefold:

1. To introduce and explain the process of pre-registration, including its significance and implementation;
2. To provide a step-by-step guide to systematic reviews and traditional meta-analyses; and
3. To offer guidance on conducting multilevel meta-analyses when assumptions of independence are not met.

### Quantitative systematic reviews

Publication rates have surged, with nearly a million articles published annually from 2016 to 2022 (Hanson et al., 2024). This overwhelming volume of literature presents significant challenges, particularly for researchers with limited time (Lima et al., 2022; Tawfik et al., 2019). For example, a psychologist studying social media would need to read through 959 articles published between 2004 and 2015 alone (Zyoud et al., 2018), making it unrealistic for a single individual to gain a comprehensive understanding of the field. Yet narrowing the scope to just a few studies risks a fragmented understanding, as singular studies often target specific populations or contexts and may yield inconsistent findings (Crocetti, 2016). Quantitative systematic reviews address these challenges by synthesizing findings from multiple studies, offering a comprehensive overview without sacrificing breadth (Lima et al., 2022; Tawfik et al., 2019). Quantitative systematic reviews also help to identify research gaps, clarify inconsistencies, and refine theoretical frameworks, ultimately advancing researchers’ understanding of complex phenomena.

A quantitative systematic review consists of two key components: a general systematic review and a meta-analysis (Chiappelli et al., 2018; Lau et al., 1997). A general systematic review begins with consolidating relevant records of a specific topic (Siddaway et al., 2019; Uman, 2011). The process includes (1) formulating a keyword search string, (2) using the formulated search string to retrieve records from databases (refer to “Step 5” of the systematic review tutorial), and (3) systematically evaluating the retrieved records based on predefined criteria (Siddaway et al., 2019; Tawfik et al., 2019). Once a final set of eligible studies has been established, researchers may proceed to meta-analysis.

Meta-analyses aggregate data from multiple studies, yielding a single quantitative effect size with enhanced precision and increased statistical power due to the larger aggregated sample size (Card, 2012; Cohn & Becker, 2003; Kepes et al., 2013; Trikalinos et al., 2008). Given that approximately one in seven published quantitative records across life and social sciences fields involves data fabrication or falsification (Heathers, 2024), traditional meta-analyses help mitigate the influence of biased studies, assuming that a sufficient number of methodologically rigorous records are included (Berrío & Kalliokoski, 2025; Egger et al., 2001), while also identifying potential inconsistencies and enabling subgroup analyses (Lee, 2018). Due to the versatility and advantages of meta-analyses, researchers, clinicians, and policymakers alike have utilized this method to investigate their respective hypotheses (Meyerowitz-Katz & Merone, 2020; Weisz et al., 2017).

**Fig. 1:**
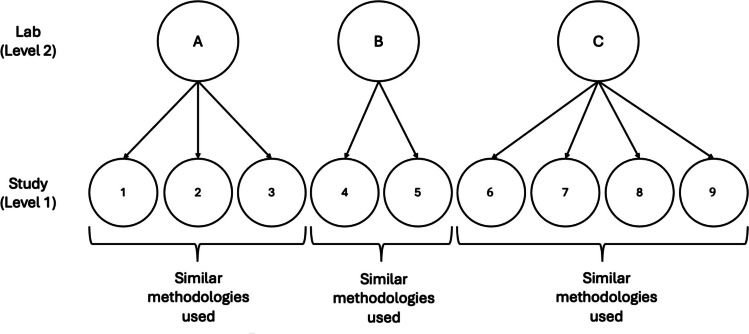
Conceptual illustration of multilevel nesting structure

Although traditional meta-analyses offer numerous advantages, they assume that effect sizes are independent, which may be violated for various reasons. For instance, studies from the same lab may share similar methodologies, which can lead to potential shared variances (e.g., experimenter bias and lab environment influences) and, consequently, non-independent effect sizes (Van den Noortgate & Onghena, 2003). In such cases, a multilevel meta-analysis may be employed (Raudenbush & Bryk, 1985). By nesting the results of the studies within the higher-level cluster of the lab from which the authors originate (e.g., Fig. 1), the non-independence of effect sizes will be accounted for.

The current tutorial is structured as follows: Firstly, we highlight the importance of pre-registering systematic reviews and meta-analyses and briefly explain the process. Secondly, we introduce systematic reviews and outline the 12 steps involved in conducting one. Thirdly, we define key terminologies of traditional and multilevel meta-analyses, explain the programming process, and interpret the results of both types of meta-analyses. While this tutorial focuses on traditional and multilevel meta-analytic techniques, we acknowledge that there are alternative methods that are beyond the scope of this paper (e.g., individual participant data meta-analysis, meta-analytic Gaussian network aggregation (MAGNA), Bayesian model-averaged meta-analysis). For readers interested in exploring these approaches, we provide recommended readings in the “Further Readings” section. Finally, we discuss the contributions and limitations of the current paper. To support this tutorial, we provide publicly available supplementary resources and *R* scripts used for the traditional and multilevel meta-analysis tutorials in an online repository (https://osf.io/dpqht). Additionally, recommended readings are provided in this tutorial for further insight.

### Pre-registration

Pre-registration involves documenting hypotheses and planned methodologies on a publicly accessible platform before data collection (Van’t Veer & Giner-Sorolla, 2016). Since systematic reviews and meta-analyses are also prone to bias introduced by data-dependent decisions, their pre-registration is as essential as that of primary studies (Sandoval-Lentisco et al., 2025). By ensuring that methodological decisions are documented ahead of time, pre-registration reduces the risk of researchers omitting or adjusting PRISMA (i.e., Preferred Reporting Items for Systematic Reviews and Meta-Analyses; Moher et al., 2009; Page et al., 2021) decision items, thus enhancing the credibility of the review. A robust pre-registration should adhere to established standards relevant to the study design (e.g., Journal Article Reporting Standards [JARS]–Quant; Appelbaum et al., 2018). The current tutorial refers to the PRISMA guidelines (refer to “Step 12: PRISMA and reporting of the systematic review in the method section” of the systematic review tutorial) which outline essential research decisions (e.g., keyword search strings and databases; refer to “Step 3: Formulating the keyword search string” of the systematic review tutorial), as well as items that are often omitted but remain important (e.g., publication bias and heterogeneity assessment methods; refer to “Tutorial 2: Traditional meta-analysis” and “Tutorial 3: Multilevel meta-analysis”; Sandoval-Lentisco et al., 2025). Platforms such as Open Science Framework (OSF) and PROSPERO are available for pre-registration (Pieper & Rombey, 2022; Simmons et al., 2021). Researchers may refer to their choice of platform for more information on the pre-registration process.

However, the pre-registered protocols may lack detailed research plans, and deviations from them may go undisclosed. Sandoval-Lentisco et al. (2025) offer numerous recommendations to enhance transparency and methodological rigor in pre-registration, which we encourage researchers to examine further. Among those recommendations, we highlight the following: Firstly, researchers may consider conducting data simulations before pre-registration to explore potential analytical choices and refine methodological decisions (for details, see Gambarota & Altoè, 2024). Secondly, any deviations from the pre-registration should be clearly disclosed and justified in the final article. One useful approach is to use deviation-disclosure tables to systematically document any changes (see Willroth & Atherton, 2024, for an example). With a detailed pre-registration, researchers can strengthen the credibility and reproducibility of their systematic reviews and meta-analyses, leading to more robust and trustworthy findings (for examples of pre-registered quantitative systematic reviews, see Kasturiratna et al., 2025 and Ong et al., 2020). Therefore, pre-registration serves as the cornerstone of open and reliable science, and we strongly encourage researchers to incorporate it in their own quantitative systematic reviews.

## Tutorial 1: Systematic review

A systematic review entails a rigorous process of identifying and collecting relevant records. Though a systematic review may be conducted without a meta-analysis, a meta-analysis typically requires a systematic review as its foundation (Bangdiwala, 2024; Khan et al., 2003). The process of conducting a systematic review includes retrieving records from various sources, followed by an evaluation of their quantity and quality (Sataloff et al., 2021). The entire process adheres to strict methodological guidelines to ensure the accuracy, transparency, reproducibility, and generalizability of the meta-analytic results (Moosapour et al., 2021; Sataloff et al., 2021). Thus, conducting a systematic review is a crucial prerequisite for meta-analysis, as it ensures that the analysis is based on a body of evidence that has been thoroughly evaluated, supporting the robustness of conclusions drawn from quantitative systematic reviews (Sohrabi et al., 2021).

In this tutorial, the PRISMA framework (Moher et al., 2009; Page et al., 2021) will be used to explain each step of the systematic review process. To enhance the clarity of the tutorial, we will be referencing a meta-analysis conducted by Majeed and colleagues (2021), which investigated the association between clinically diagnosed dyslexia and creativity. Due to conflicting findings in the literature, the authors aimed to determine whether these mixed findings stem from the methodological differences across studies.

### Step 1: Crafting a research question

The key to a meaningful systematic review is a clearly defined research question, which guides subsequent steps and clarifies key aspects of the data collection process (e.g., determining which studies to include). One commonly used framework is PICO (Participant, Intervention, Control, Outcome), as outlined in Table 1 (Hosseini et al., 2024; Richardson et al., 1995). To create our example research question, “What is the association between clinically diagnosed dyslexia and creativity?”, the following PICO framework could have been utilized (see Table 1). Alternatively, researchers may utilize the FINER (Feasible, Interesting, Novel, Ethical, and Relevant; Fandino, 2019; Willis, 2023) criteria to create their research questions. Further details on the FINER framework are available in the supplementary document in the current work’s associated OSF.

**Table 1:** Overview of forming the research question and inclusion and exclusion criteria using the PICO framework

			Inclusion/exclusion criteria^a^	
PICO component	Definition	Components of research question	Title and abstract screening	Full-text screening
Population	The population or group of individuals of interest to which the findings will generalize to	All humans	Records were included if the sample was human. No restrictions were applied to the sample characteristics, such as age	Records were included if the sample was human. No restrictions were applied to the sample characteristics, such as age
Intervention	The treatment of interest (for clinical and intervention-based studies) or the study variable that influences the outcome variables (for correlational studies)	Clinically diagnosed with dyslexia	Records were included if dyslexia was mentioned	Records were included if dyslexia was clinically diagnosed, such as by a psychologist or through a medical center
Comparator	The alternative to the intervention being evaluated (for clinical and intervention-based studies)	Controls/Not clinically diagnosed with dyslexia	Not applicable	Records were included if at least one healthy (i.e., not diagnosed with dyslexia) control group was available
Outcome	The effects or results of the intervention being investigated	Creativity	Records were included if creativity or divergent thinking was mentioned Records were included if it was an empirical and quantitative study	Records were included if creativity was explicitly studied through a creativity task, such as a divergent thinking task Records were included if there was adequate quantitative information or statistics that allowed the effect sizes to be computed. If a study did not have sufficient information, relevant authors will be contacted via email, ResearchGate, or any communication channels

*Note.* A language criterion should be included in both the title and abstract screening, as well as the full-text screening stages (e.g., “Record was published in English, Chinese, Malay, or Bahasa Indonesia.”). Records should be in languages the researchers are fluent in, unless researchers have the means to accurately translate the paper. While some systematic reviews limit inclusion to English-language records to save time and resources (Dobrescu et al., 2021), this approach may reduce generalizability by over-representing WEIRD (Western, Educated, Industrialized, Rich, Democratic) populations, which have been shown to differ from other populations (Henrich et al., 2010a, b)

^a^Refer to “Step 6: Inclusion/exclusion criteria” of the systematic review tutorial for more details

### Step 2: Choosing databases

To systematically identify relevant literature aligned with the research question, researchers may conduct database searches in relevant sources and supplement these with hand searches. It is recommended that at least two databases be selected to ensure a thorough identification of records (Ewald et al., 2022), as different databases cover different types of literature (e.g., peer-reviewed journal articles versus doctoral dissertations; Harari et al., 2020) and different sources within the literature (e.g., different journals; McDonald et al., 1999). The choice of databases should align with the researcher’s research question and/or topic. For example, *CINAHL* is typically used for nursing and allied health-related research, while *ABI*/*Inform Complete* is used for business-related research. If available, researchers may refer to their institutional library’s database repository to review and determine which databases best align with their research question, or request assistance from their institutional librarians.

Within psychology, we recommend the following databases: *Semantic Scholar* (covers all fields of science), *PsycINFO* (focuses on psychological literature), *PubMed* (focuses on biomedical and life sciences research, including health and cognitive psychology), and *Web of Science Core Collection* and *Scopus* (covering multidisciplinary research). These databases were chosen for their comprehensive indexing and relevance to psychological research. Alternatively, researchers may utilize the publicly available program, *Publish or Perish*, to search through multiple databases (e.g., *Scopus*, *Web of Science Core Collection*, *PubMed*) for literature.^1^ It is important to note that some databases may require a paid subscription, which might be covered by academic institutions.

Researchers should also conduct a hand search, which refers to the manual process of reviewing specific journals that are relevant to the research question and/or topic, and other sources (e.g., grey literature, which are unpublished papers that are not peer-reviewed) to identify relevant records that may not be indexed in databases (Higgins et al., 2024). Additionally, forward citation searching (identifying papers that cited the retrieved record, especially if they are seminal works) and backward citation searching (reviewing the references cited within the retrieved record) may be employed by the researchers to capture more relevant studies. Specifically, the inclusion of grey literature is crucial for mitigating publication bias, as significant findings are often favored over non-significant ones, potentially resulting in exaggerated results that may misrepresent the true nature of the topic (Bartoš et al., 2023; Siddaway et al., 2019). Furthermore, grey literature includes research that has not yet been formally published (e.g., preprints and dissertations). Although such works may not be indexed in databases, they are still important to include, as they often provide up-to-date and cutting-edge research findings. We recommend *Google Scholar*, *PsyArXiv*, and *ProQuest Dissertations & Theses Global* as sources for grey literature due to their accessibility, scope, and extensive coverage of academic materials (Paez, 2017; Rothstein & Hopewell, 2009; Skubera et al., 2025).

### Step 3: Formulating the keyword search string

A search string should be crafted to capture relevant records. Developing a well-crafted search string involves several key considerations. Firstly, researchers should identify key concepts of the research question. This includes identifying the core themes and synonyms related to the core themes. In our example, the core themes are dyslexia and creativity. Therefore, words or phrases that constitute dyslexia and creativity should be included (for more information, refer to Table 2).

**Table 2:** Examples of key concepts of the research question

Core Themes	Synonyms	Measurements	Factors
Dyslexia	“Word blindness”	NIL	NIL
Creativity	“Divergent thinking”^a^	Creativity Assessment Packet Test	General creativity or a category of creativity (e.g., divergent thinking)

^a^The key concepts included in the table were terms utilized by the original authors (Majeed et al., 2021). Researchers interested in literature surrounding creativity may consider related synonyms such as “imagination”, “innovative”, and “ideation” if they intend to study broader aspects of creativity.

Secondly, the use of search operators (Table 3) is an effective way to refine or expand search results, but must be applied carefully to avoid missing relevant records. For example, by using the Boolean operator “NOT” in “dyslexia NOT ADHD”, the database will exclude papers that mention “ADHD”, even if the papers contain relevant information on “dyslexia”. Wildcards, truncations, and quotation marks may also be used, but with caution, as their functionality varies across the different databases (e.g., *Google Scholar* does not support the truncation operator; Bramer et al., 2017), and the meaning of these symbols may differ across databases. Therefore, it is important to consult each database’s syntax guide and adjust the syntax accordingly.

**Table 3:** Overview of search operators

Type	Purpose	Examples
Wildcards	Represent specific unknown characters in a word	“synthesi?e” could return “synthesize” and “synthesise”
Truncation	Capture different word variations that share the same root word by adding a symbol at the end	creativ* retrieves creative, creativity, and creatively
Quotation marks	Capture a phrase in the specific order	Using “reading disorder” instead of using “reading” and “disorder” as separate keywords
Boolean operator	Expand or specify the search results	“AND”, “OR”, and “NOT” to specify, expand, or exclude certain phrases, respectively

### Step 4: Conducting the literature search

Upon finalizing the keyword search string, a pilot search should be conducted in the relevant databases to ensure that sufficient literature exists on the topic of interest (Eysenbach et al., 2001). A useful guide to follow is that the pilot search should aim to verify that the search strategy is sufficiently comprehensive to capture seminal or benchmarking studies relevant to the research question (Ahmed & Rafiq, 1998; Bramer et al., 2018; Ngcobo & Zhandire, 2025; Siddaway et al., 2019) and yield an adequate number of records (e.g., 10 or more; Myung, 2023) to assess its effectiveness meaningfully. The researchers should determine the maximum number of records, though it is important to keep in mind that this is only a pilot search. Additionally, researchers are encouraged to review the initial search results to assess their relevance to the research question and to ensure that seminal or benchmarking studies are captured, thereby confirming that the search strategy is sufficiently comprehensive (Siddaway et al., 2019). After completing the pilot, researchers may begin conducting their literature search using their selected databases. When conducting a hand search in *Google Scholar*, it is advised to include the first 200–300 records to ensure a comprehensive coverage of both published and grey literature (Haddaway et al., 2015). For other sources of grey literature, researchers should standardize the total number of records to be screened, systematically review all the records, and focus on selecting records directly relevant to the topic of interest.

During the literature search, researchers should specify which metadata fields are being searched (e.g., title, abstract, or author keywords), as these decisions directly affect search precision and recall. For example, restricting a search to the title field implies that only articles in which the specified terms appear in the title will be retrieved. Researchers should determine which metadata fields to include by considering where the specified terms are most likely to appear (e.g., in the title, abstract, or keywords; Gusenbauer & Gauster, 2025). Furthermore, it is advisable to consult a database’s thesaurus (e.g., *APA Thesaurus of Psychological Index Terms* in *PsycINFO* or MeSH terms in *PubMed*) to identify standardized subject headings relevant to the research question. Using these controlled vocabulary terms improves retrieval accuracy by grouping related concepts under consistent subject headings (Bramer et al., 2018; Jenuwine & Floyd, 2004; Leblanc et al., 2024), ensuring that the search process remains both comprehensive and reproducible.

Additionally, researchers may employ database filters (e.g., publication date ranges, document types, language) to further enhance search precision and reduce the number of irrelevant articles retrieved. Importantly, the use of filters is best treated as a methodological decision made at the study design stage rather than applied reactively during screening. For example, restricting records to those published after the introduction of a specific intervention or technology may be appropriate when earlier records could not have meaningfully engaged with that technology. Given that such filters may also unintentionally exclude pertinent records, such filters should be used cautiously and only when theoretically justified.

### Step 5: Retrieval of records

To efficiently manage the records identified in “Step 4: Conducting the literature search”, we recommend using a reference manager such as Zotero or Mendeley. A reference manager helps store all retrieved records in one place, making them easier to access and review in subsequent steps. For example, in “Step 8: Title and abstract screening”, having all abstracts readily available within a reference manager, instead of manually searching for them individually, may save a significant amount of time.

While retrieving the records from the databases, researchers should ensure that the downloaded records are in .ris format, as this format is widely supported by most reference management tools.^2^ After downloading the files, researchers may use a reference manager to store and organize the downloaded files by importing them into the software. Duplicate records should then be merged or removed.^3^ After completing the literature search and retrieval, researchers should report the search and retrieval date clearly in the methods section of the manuscript (e.g., “A systematic search was conducted on 20 November 2024 to search for relevant literature”). Documenting the search and retrieval date is necessary for researchers to track when the retrieval was conducted. If one year has passed since the original retrieval, and the paper has yet to be published, an update of the search may be necessary (Lefebvre et al., 2019). This practice will help ensure that the included records are relevant and up-to-date. Researchers may refer to the current work’s associated OSF for a template to comprehensively document the retrieval process. Finally, researchers should consider conducting a new or updated systematic review or meta-analysis when new evidence or methodological advances could significantly alter the conclusions of the original meta-analysis (Bashir et al., 2018; Garner et al., 2016; Siddaway et al., 2019).

### Step 6: Inclusion/exclusion criteria

Establishing well-defined inclusion and exclusion criteria is imperative for the systematic screening and selection of retrieved records in later steps. When formulating the criteria, researchers can reference the frameworks introduced in “Step 1: Crafting a research question”. For instance, if the PICO framework was used when formulating the research question (Booth et al., 2015), the same framework can be applied to structure the inclusion and exclusion criteria. To illustrate an example of the inclusion criteria, we referenced the PICO framework and research question outlined previously (“What is the association between clinically diagnosed dyslexia and creativity?”; Table 1). Furthermore, given that two screening stages will be conducted, it is necessary to establish distinct inclusion criteria for each. The full-text screening stage should apply the aforementioned comprehensive set of criteria. In contrast, the title and abstract screening stage should employ a simplified version, appropriate to the limited information available in these sections. Table 1 compares the inclusion criteria for each stage, demonstrating how they are adapted to reflect the amount of information available at each screening stage.

### Step 7: Inter-rater reliability/agreement

Before proceeding with the subsequent steps, it is important to understand inter-rater reliability/agreement (IRR/IRA), which refers to the consistency or agreement of ratings between the reviewers. There are multiple methods to calculate IRR/IRA, such as percent agreement and Cohen’s κ, and acceptable cutoff values differ depending on the index used (Lombard et al., 2002; McHugh, 2012). For more information on the calculation procedures for the percent agreement and Cohen’s κ methods, researchers may refer to the supplementary document in the current work’s associated OSF. Researchers should note that the selection of the most appropriate measure of IRR/IRA depends on the complexity of the coding decisions, the number of coders, and the type of data being coded (e.g., nominal, ordinal; Cole, 2024). Importantly, the rationale for the index chosen should be clearly justified and reported (Lombard et al., 2002).

When screening papers and conducting quality assessment (i.e., Steps 8, 9, 10), at least two reviewers should be assigned to evaluate each study according to the inclusion and exclusion criteria. The reviewers can be trained research assistants (RAs), the researchers involved in the project, or a mix of both. During the screening process, reviewers should screen independently (i.e., without any discussion) to avoid influencing each other’s opinions. Additionally, they must share a common understanding of the screening criteria to ensure that papers are not mistakenly included or excluded during the screening process. One plausible way to ensure there is alignment between reviewers is to conduct a briefing session prior to the screening. Alternatively, reviewers may use a trial set, in which they screen a subset of the total number of records (e.g., 10 out of 50), typically comprising 10–20% of the total number of records (McDonagh et al., 2013). However, the final number of records for the trial set should still be dependent on multiple factors (e.g., the total number of records, the complexity of the research question). If the size of the trial set is too large, it may resemble a full screening and delay necessary refinements to the criteria. Conversely, a trial set that is too small may yield a disproportionately high or low IRR or IRA that is not reflective of the reviewers’ screening ability.

It is advisable to resolve and clarify any discrepancies between reviewers arising from misinterpretations of the criteria during the trial phase of the subsequent steps (e.g., screening and quality assessment). Generally, an IRR/IRA value of 60% or higher is considered acceptable before proceeding (McHugh, 2012). Once researchers begin the actual screening or quality assessment, an IRR/IRA value of approximately 80% or higher is often considered acceptable before continuing to the next stage of the systematic review process (Belur et al., 2021; Lombard et al., 2002). However, these thresholds should not be treated as universal cutoffs. Rather, these cutoff values serve as reference points that prompt further discussion among reviewers regarding sources of disagreement and potential refinements to the inclusion/exclusion criteria, thus enhancing the consistency of studies included in the systematic review or meta-analysis (Cole, 2024). Researchers should still select the appropriate minimum acceptable level of reliability for the choice of index used (Lombard et al., 2002). Higher levels of IRR/IRA should be used for the percent agreement method as it does not take into account agreement that may happen by chance and thus overestimates reliability (Lombard et al., 2002). Conversely, lower levels of IRR/IRA for more complex indices (e.g., Cohen’s κ) may be acceptable as it accounts for chance agreement and therefore, more conservative (Lombard et al., 2002).

While IRR/IRA is especially relevant during stages such as screening, its usability for multi-categorical decisions (e.g., risk of bias, quality assessment, or data extraction) is more complex. For simplicity, researchers conducting these steps may still apply a form of percentage agreement by treating the comparison as a binary match or mismatch (e.g., whether reviewers assigned the same quality rating during the quality assessment or extracted the same data elements during the data extraction). However, this approach may be less suitable when judgments involve a rating scale or qualitative interpretation, where partial agreement is possible. When reviewers have to provide ordinal judgements, such as risk of bias or quality assessment ratings (e.g., low, moderate, high), researchers may use weighted κ (Li et al., 2023). Beyond categorical ratings, when reviewers extract quantitative data that may vary slightly due to calculation, for instance, effect sizes derived from reported statistics, or other continuous outcomes, the intraclass correlation coefficient (ICC) may be more appropriate (Koo & Li, 2016), as it evaluates the consistency of quantitative measurements between reviewers. Overall, the purpose of assessing IRR/IRA is to ensure that reviewer decisions are made consistently and transparently across the applicable stages of the systematic review process, thereby strengthening the methodological rigor and reproducibility of the review (Belur et al., 2021; Siddaway et al., 2019).

### Step 8: Title and abstract screening

After completing Steps 1 to 6, researchers can proceed to screen the retrieved records based on their title and abstract. This step significantly reduces the number of records retrieved, streamlining the subsequent steps and allowing researchers to proceed more efficiently (Polanin et al., 2019). There are two main approaches to this stage:

1. Screening titles first, followed by abstracts, or
2. Screening titles and abstracts together.

While the first approach may enable the efficiency of subsequent steps by reducing the number of irrelevant records to screen, it also increases the risk of overlooking relevant records (for more information, see Mateen et al., 2013; Teo et al., 2023). Therefore, the second approach is generally preferred, as it has been shown to identify a larger number of relevant records and thus maximize the comprehensiveness of the review (Teo et al., 2023).

Before screening the records, the .ris and .nbib files of the retrieved records (i.e., “Step 5: Retrieval of records”) should be exported from the reference manager into a .csv format. The .csv file can then be opened directly in spreadsheet software such as Google Sheets or Microsoft Excel. Within the .csv file, columns should be created to facilitate the screening process, with the number of columns corresponding to the number of inclusion criteria (refer to Table 1 for the inclusion criteria applied at this stage). For each criterion, researchers should indicate their screening decision by stating “yes”, “no” or “unclear” in the corresponding cell. If all criteria are marked as either “yes” or “unclear”, the record should be included for the next stage of screening (i.e., “Step 9: Full-text screening”). Including the “unclear” option may help to prevent the exclusion of potentially relevant papers as detailed information may only be available during the full-text screening stage. However, the “unclear” option should be used sparingly, as it may unnecessarily increase the number of full-text articles to be screened, potentially wasting time and resources (Polanin et al., 2019).

**Fig. 2:**
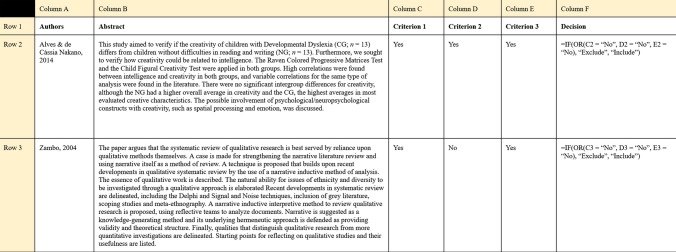
Example spreadsheet for title and abstract screening stage

Additionally, a decision column should be added to indicate which records will proceed to “Step 9: Full-text screening”. Papers that meet all the criteria can be marked as “include” in the decision column while papers that failed any of the criteria can be marked as “exclude”— the process of which can be automated using a formula, helping to minimize human error. Once the title and abstract screening sheet has been completed, researchers can begin the inter-rater process (i.e., “Step 7: Inter-rater reliability/agreement”) and resolve any discrepancies. If the reviewers are unable to reach a consensus, two options may be considered: A third reviewer may resolve the discrepancy, or otherwise, the record may be automatically advanced for further screening. However, the latter risks include a high quantity of irrelevant papers, so the decision requires careful consideration. All records marked as “include” in the decision column will proceed for further screening at the full-text stage. Figure 2 provides a sample spreadsheet that may facilitate the title and abstract screening process, along with an example formula for automating the decision column.

### Step 9: Full-text screening

At this stage, records that meet the eligibility criteria in “Step 8: Title and abstract screening” will undergo further screening. When full-text articles are not readily accessible, researchers are encouraged to search for author-uploaded versions on ResearchGate, contact the corresponding author(s) to request a copy, or submit an Interlibrary Loan (ILL) request via their institution’s library services.

The full set of inclusion/exclusion criteria from “Step 6: Inclusion/exclusion criteria” will be utilized for the full-text screening. IRR/IRA should also be calculated at this step, and any discrepancies should be resolved between the reviewers or with the involvement of a third reviewer. For more information on how to calculate IRR/IRA, readers may refer to “Step 7: Inter-rater reliability/agreement”.

### Step 10: Assessment of quality of evidence

Following “Step 9: Full-text screening”, the next step typically involves assessing the quality of evidence in the papers included. We recommend using the GRADE (Grading of Recommendations, Assessment, Development and Evaluation) framework for this evaluation (Balshem et al., 2011). To ensure reliability and minimize bias, it is recommended to have at least two reviewers independently engage in this assessment. The process begins by identifying the relevant outcomes of the study (e.g., creativity). Certainty of evidence is rated on a four-level scale: very low, low, moderate, or high. The initial rating depends on the study design: randomized controlled trials start as high certainty, while non-randomized controlled trials start as low. The certainty can then be downgraded based on five domains: risk of bias, inconsistency (e.g., heterogeneity in results across studies), indirectness (e.g., differences between the sample used and the target population), imprecision (e.g., small sample sizes), and publication bias. The inconsistency, imprecision, and publication bias domains are elaborated in the subsequent meta-analysis section, while the risk of bias domain is expanded upon in the following paragraph. Indirectness may be assessed based on how generalizable the methodology and results of each paper are. For observational studies, the certainty can be upgraded if there is a large magnitude of effect, evidence of a dose-response relationship, or inclusion of plausible confounds. Finally, these ratings should be incorporated into the methods and discussion sections of the paper. Including GRADE helps ensure consistency and transparency of the evidence included (Al Duhailib et al., 2024; Moberg et al., 2018), and researchers interested in the GRADE framework may refer to the “Further readings” section for more information.

Risk of bias in the included studies is evaluated using tools appropriate to the study design (Page et al., 2021). To date, no standardized tool has been established for assessing bias in studies with continuous variables, but several tools are available for evaluating bias in studies with categorical variables (Table 4). Researchers may also consider alternative risk of bias (RoB) or quality assessment (QA) instruments such as RoB, RoB 2, ROBINS-I, and ROBUST-RCT (Higgins et al., 2011; Sterne et al., 2016; Sterne et al., 2019; Wang et al., 2025). Regardless of the selected tool, it is recommended that at least two reviewers independently appraise each included study. Afterwards, the IRR/IRA should be calculated (refer to “Step 7: Inter-rater reliability/agreement”), and any discrepancies should be resolved among the reviewers or with a third reviewer. If a study was found to be biased, researchers may consider excluding the specific study in their analysis (Bero, 2025). In this example, the most suitable tool is the Newcastle–Ottawa Scale (Wells et al., 2000) as the included studies were non-randomized, with the groups consisting of people who were diagnosed with dyslexia and those who were not. Table 4 compares the Cochrane Risk of Bias Tool and the Newcastle–Ottawa Scale, highlighting the rationale for using the latter in this example.

**Table 4:** Elaboration of risk of bias tools

	Cochrane Risk of Bias Tool	Newcastle–Ottawa Scale
When to use	Randomized studies (i.e., cluster-randomized, randomized controlled, and cross-over trials; Higgins et al., 2024; Sterne et al., 2019)	Non-randomized studies (i.e., cohort and case–control studies; Wells et al., 2000)
Assessment domains	(1) Randomization process (2) Abnormalities from the interventions (3) Missing data (4) Measurement of the outcome (5) Selection of the reported findings	(1) Selection of sample (cohort studies) and cases (case–control studies) (2) Comparability (i.e., whether the sample or cohort matches the study design) (3) Assessment of outcome (cohort studies) or exposure (case–control studies)
Scoring	Record their responses and justification for each domain based on “Low risk of bias”, “High risk of bias”, or “Some concerns”	A maximum of one star can be awarded for each item question for the selection and assessment of outcome or exposure domain A maximum of two stars can be awarded to the one-item question for comparability domain

### Step 11: Data extraction

**Fig. 3:**
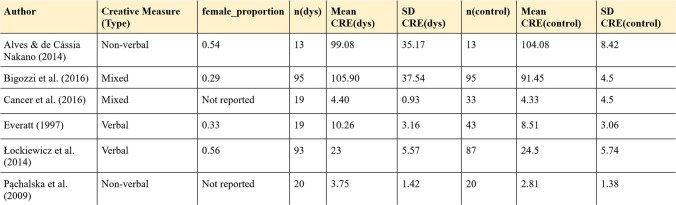
Format for data extraction table

After completing Step 10, the researchers can proceed to extract relevant information from the finalized set of studies. As with the aforementioned steps (i.e., Steps 8, 9, and 10), data extraction should be conducted independently by two reviewers. Afterwards, IRR/IRA should be computed (i.e., “Step 7: Inter-rater reliability/agreement”), and any discrepancies identified should be resolved through discussion between the reviewers or, if necessary, with a third reviewer. Additionally, during the extraction process, reviewers should ensure that the included studies are not retracted (i.e., previously published articles being withdrawn from the journal). Retracted records may contain unreliable information that may influence the results of the meta-analysis (Graña Possamai et al., 2025). Figure 3 provides a sample format of the data extraction table.

### Step 12: PRISMA and reporting of the systematic review in the method section

**Fig. 4:**
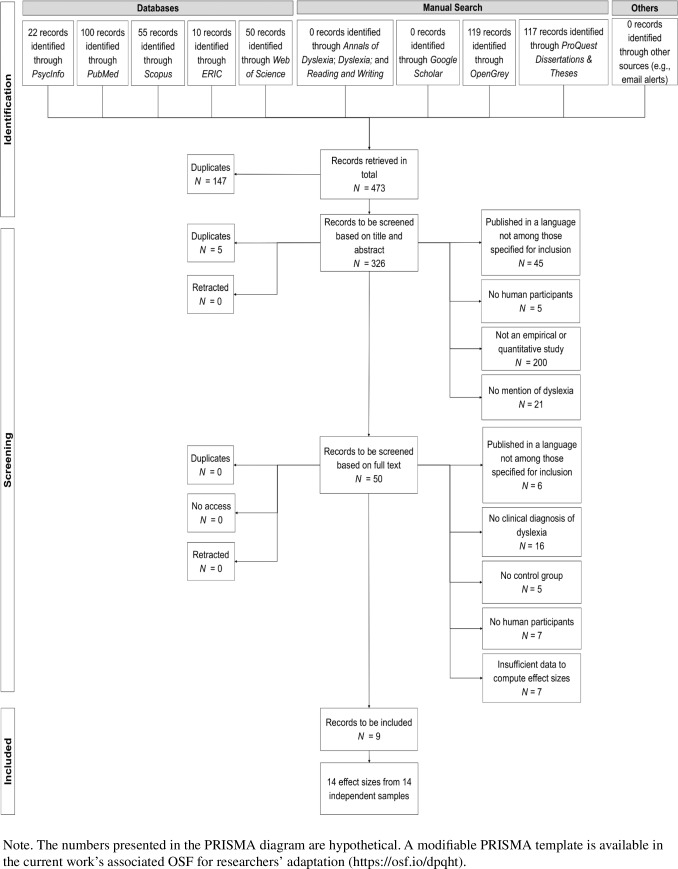
Example of a PRISMA diagram

With all methodological steps completed, the systematic review process should be reported clearly, systematically, and logically in the manuscript’s methods section to ensure transparency, reproducibility, and ease of understanding. One of the best practices for presenting this information is to include a PRISMA flow diagram (e.g., Fig. 4), which visually summarizes the entire review process, from the initial identification and retrieval of records to the final number of studies included for synthesis (Moher et al., 2009; Page et al., 2021). The diagram should be organized into three key components^4^: identification, screening, and inclusion (Siddaway et al., 2019) and is typically used to present information such as the databases and other sources consulted (e.g., journals), the search terms applied, the time frame of the literature search, and the number of records at each stage of the screening process. In addition to the diagram, the methods section should also specify the inclusion and exclusion criteria and report inter-rater agreement for title and abstract screening, full-text screening, and data extraction. Together, the PRISMA diagram and accompanying methodological details provide a comprehensive and transparent account of the review process, enabling readers to critically appraise the rigor of the study and replicate its procedures if needed. With the systematic review completed, the following sections will outline the procedure for conducting a meta-analysis.

### Statistical concepts of meta-analysis

Having completed the systematic review, researchers can proceed to synthesize the collected effect sizes through meta-analysis. To lay the groundwork, the following section outlines the programming tools and core statistical concepts necessary for understanding and conducting meta-analytic techniques.

### Statistical programming

For the traditional and multilevel meta-analysis tutorials, we will utilize *R* version 4.5.0 (R Core Team, 2025), an open-source statistical programming language, to ensure accessibility, cost-effectiveness, and transparency. The open-source nature of *R* facilitates reproducibility and openness in research, making it a practical choice for conducting meta-analyses. Several *R* packages support meta-analysis (Polanin et al., 2017), with three popular ones being *meta* (Balduzzi et al., 2019), *metafor* (Viechtbauer, 2010), and *metaSEM* (Cheung, 2015a). The *meta* package is well-suited for most meta-analyses and is user-friendly as it requires minimal programming (Lortie & Filazzola, 2020). However, the dataset would be required to have pre-calculated effect sizes that the *meta* package is unable to generate from study-level summary statistics such as means and standard deviations (Lortie & Filazzola, 2020). In contrast, the *metafor* package allows researchers to compute the various effect sizes (e.g., standardized mean differences) directly from each study's summary statistics (Lortie & Filazzola, 2020). Furthermore, the *metafor* package provides comprehensive tools for more complex meta-analyses, such as random-effects models, multilevel modelling, and inclusion of moderation (Polanin et al., 2017). Lastly, the *metaSEM* package allows for complex meta-analyses by employing a structural equation modelling approach (SEM; Cheung, 2015a; Polanin et al., 2017), though it is not used in this tutorial. Researchers interested in SEM-based meta-analysis may refer to the “Further Readings” section for more information.

In this tutorial, we will thus be utilizing the *metafor* package version from 4.6-0.6 to 4.8-0 (Viechtbauer, 2010) to facilitate effect size computation and support advanced analyses (i.e., multilevel meta-analyses). All *R* scripts and example data used are available on the current work’s associated OSF (https://osf.io/dpqht).

### Terminologies

Researchers should have a strong foundation in fundamental statistical concepts such as statistical power, confidence intervals (CIs), and moderators to fully understand the material (for more information, see Kaplan et al., 2020, and Vandever, 2020). However, to facilitate a deeper comprehension of the meta-analysis script, we will also clarify key terminologies that may be less widely known below (i.e., effect sizes, measures of heterogeneity, and meta-analytic models).

#### Effect sizes

Effect size refers to the strength of a relationship between variables (Borenstein et al., 2021; Kelley & Preacher, 2012). Statistical significance, which used to be the sole proof of significant results (Hayat, 2010), is potentially misleading as it depends on several factors such as the number of participants in the sample and the statistical test being employed (Sullivan et al., 2012). Hence, effect sizes help clarify the magnitude of the findings, while statistical significance indicates how likely the results were due to chance (Aarts et al., 2014). To calculate effect size, it is important to note that true effect size is not synonymous with the observed effect size as found in published studies (Harrer et al., 2021). This discrepancy arises due to sampling error, which occurs as each primary study can only be conducted with a relatively small sample size drawn from the population to which the results are intended to be generalized to (Harrer et al., 2021). The formula relating an observed effect size ${\widehat{\theta }}_{k}$ to the true effect size ${\theta }_{k}$ of a study *k* is as follows:

$${\widehat{\theta }}_{k}= {\theta }_{k}+{\varepsilon }_{k}$$

where ${\varepsilon }_{k}$ represents the sampling error of study *k*.

The most common measures of effect sizes are the Pearson correlation coefficient *r* (Galton, 1889; Pearson, 1896), Cohen’s *d* (Cohen, 1988), Hedges’ *g* (Hedges, 1981), odds ratio (Cornfield, 1951), and relative risk/risk ratio (Cornfield, 1951). The choice of effect size for a meta-analysis depends on the type of data and the research question. In cases where included records report different effect sizes for the same phenomenon of interest, researchers may convert effect sizes to a common metric (Borenstein et al., 2021; Chinn, 2000; Valentine et al., 2019; Jacobs & Viechtbauer, 2017; Polanin & Snilstveit, 2016; Villacura-Herrera & Kenner, 2020). The *effectsize* package in *R* (Ben-Shachar et al., 2020) provides a detailed description of performing such conversions for various effect sizes. For example, the *d_to_r* function can be used to convert a Cohen’s *d* effect size to a correlation *r*, while the *r_to_oddsratio* function allows for the conversion of Pearson *r* into an odds ratio. Researchers who are interested in the conversion of effect sizes may refer to articles listed in the “Further Readings” section for more information. Table 5 provides an overview of the different types of effect sizes (Ellis, 2010) along with corresponding example research questions.

**Table 5:** Types of effect sizes

Index	Description	Suitable for	General guideline	Example
Pearson’s *r*	Quantifies the strength and direction of a relationship between two variables; Correlation index	Continuous variables	As recommended by Funder and Ozer (2019), .05 to .10 represents a very small effect, .10 to .20 represents a small effect, .20 to .30 represents a medium effect, .30 to .40 represents a large effect, and values greater than .40 represent a very large effect	What is the relationship between the Big 5 variables and prosociality?
Cohen’s *d*	Difference between two group means	Continuous variables; Large sample sizes	As recommended by Cohen (1988), 0.20 to 0.50 represents a small effect, 0.50 to 0.80 represents a medium effect, and more than 0.80 represents a large effect	Do men and women differ in social media usage?
Hedges’ *g*	Difference between two group means	Continuous variables; Mix of small and large sample sizes	As recommended by Cohen (1988), 0.20 to 0.50 represents a small effect, 0.50 to 0.80 represents a medium effect, and more than 0.80 represents a large effect	Do men and women differ in social media usage?
Odds ratio (OR)	Comparison of the odds of an event occurring in one group to another	Dichotomous outcomes; Case-control studies	It is recommended to convert the odds ratio to another index of effect size (e.g., risk ratio) for interpretation as the value may be unintuitive (O’Connor, 2013; Persoskie and Ferrer, 2017) An OR of 1 indicates no association between exposure and outcome. An OR greater than 1 suggests higher odds of association with the exposure and outcome. An OR less than 1 suggests lower odds of association between exposure and outcome.	How likely was performance improvement given that the employee underwent training?
Risk ratio (RR)	Comparison of the probability of an event occurring in one group to another	Dichotomous outcomes; Randomized controlled trials and cohort studies	A risk ratio of 1 suggests no difference in probability of the event in either group, more than 1 suggests that the higher probability of the event is in the treatment group as compared to the control group, and less than 1 suggests a lower probability of the event in the treatment group as compared to the control group	How likely was performance improvement in employees who underwent training?

#### Measures of heterogeneity

In a meta-analysis, the studies included will differ in methodology to a certain extent. This difference, known as heterogeneity, has to be quantified as it may affect the results and interpretations of the meta-analysis (Higgins & Thompson, 2002). Common measures of heterogeneity include Cochran’s *Q* (Cochran, 1954) and the statistic ${I}^{2}$ (Higgins & Thompson, 2002). Cochran’s *Q* is used to distinguish the studies’ sampling error from actual between-study heterogeneity (for more information, refer to Cochran, 1954 and Harrer et al., 2021). A significant *Q* test indicates potential moderators that are not accounted for in the meta-analytic model specified (Hedges & Olkin, 1985). However, the significance of *Q* is highly dependent on the size and statistical power of the meta-analysis, making it less able to assess heterogeneity in large datasets (Harrer et al., 2021).

*Q* is calculated as:

$$Q = {\sum }_{k = 1}^{K}\frac{1}{Var({\widehat{\theta }}_{k})}{({\widehat{\theta }}_{k}- \widehat{\theta })}^{2}$$

whereby *Q* uses the deviation of each study’s observed effect ${\widehat{\theta }}_{k}$ from the summary effect $\widehat{\theta }$, weighted by the inverse of the study’s variance, $\frac{1}{Var({\widehat{\theta }}_{k})}$, and *K* represents the total number of studies.

An alternative assessment of heterogeneity would be the ${I}^{2}$ statistic, which indicates the percentage of variation in the effect sizes that is attributed to the true heterogeneity across studies (Higgins & Thompson, 2002) and is based on Cochran’s *Q.* The formula for ${I}^{2}$ is as follows:

$${I}^{2 }= \frac{Q - (K-1)}{Q}$$

Researchers should note that negative values of ${I}^{2}$ are not theoretically plausible and should therefore be set to zero (Harrer et al., 2021). To aid interpretation of the values of ${I}^{2}$ researchers may follow these guidelines: 25%, 50%, and 75% represent low, moderate and high heterogeneity respectively (Borenstein et al., 2021).

Cochran’s *Q* test only assesses the statistical significance of heterogeneity, while Higgins and Thompson’s ${I}^{2}$ indicates the magnitude of heterogeneity (Huedo-Medina et al., 2006). Given that *Q* is highly dependent on the size and statistical power of the meta-analysis (Harrer et al., 2021), ${I}^{2}$ is often preferred as a more informative measure of heterogeneity. We recommend reporting both statistics, as ${I}^{2}$ complements the *Q* test by providing a clearer indication of the presence and magnitude of heterogeneity in a meta-analysis (Huedo-Medina et al., 2006).

#### Meta-analytic models

Firstly, the random-effects model assumes that the underlying effect size varies across studies in a meta-analysis. The variation may be due to differences across the samples or of the methodologies used in the studies (Dettori et al., 2022). Hence, if at least one of the conditions is met:

1. When there is an assumption of heterogeneity between studies, or
2. When between-study variability is important, a random-effects model is utilized.^5^

To allow for hypothesis testing of the research question, the random effects are assumed to be normally distributed. There are several methods to estimate the between-study variance in a random-effects model (e.g., DerSimonian and Laird, Mandel-Paule, Sidik and Jonkman).^6^ For the examples used in the current tutorials, the restricted maximum likelihood (REML) estimation will be used due to its reduced bias in variance components (Novianti et al., 2014; Veroniki et al., 2016; Viechtbauer, 2005).

Secondly, the fixed-effects model refers to the assumption that all the studies included in the meta-analysis are estimating the same effect sizes. Any observed differences are assumed to be due to sampling error rather than the true differences in effect sizes. In such cases, fixed-effects models are used under the assumption of homogeneity across studies.

Lastly, the mixed-effects model consists of aspects of both a random-effects model and a fixed-effects model. Variation between study results is attributed to both random and fixed variables (Hedges, 1992). Thus, recommendations of the between-study variance estimations can be similarly applied to mixed-effects models. The mixed-effects model can also consist of at least one moderator (Jain et al., 2019). Refer to the document in the current work’s associated OSF for examples of research questions aligned with the type of modelling used, and their corresponding assumptions required.

## Tutorial 2: Traditional meta-analysis

In this tutorial, we will be using data from the meta-analysis conducted by Majeed and colleagues (2021), which looks at the question: “Do individuals with clinically diagnosed dyslexia exhibit higher creativity than controls without clinically diagnosed dyslexia?” The complete annotated *R* script for this tutorial is available in the current work’s associated OSF. Additionally, researchers may refer to the *R* Markdown file for a detailed explanation of the *R* script used in this tutorial.

### Programming

The dataset for a traditional meta-analysis can be in any format so long as each row contains information of one unique study (for more information on the different types of data structure, refer to the supplementary document in the current work’s associated OSF). For traditional meta-analysis, the function used for analysis is *rma*. The information in the first section of the script will differ depending on the measure of effect size and the type of variables. For example, when synthesizing group differences with quantitative variables, if the intended measure is the raw mean difference, only the means need to be computed. For more information on the types of effect sizes and their corresponding variables, refer to the article by Viechtbauer (2010) on the *metafor* package. 

**Fig. 5:**
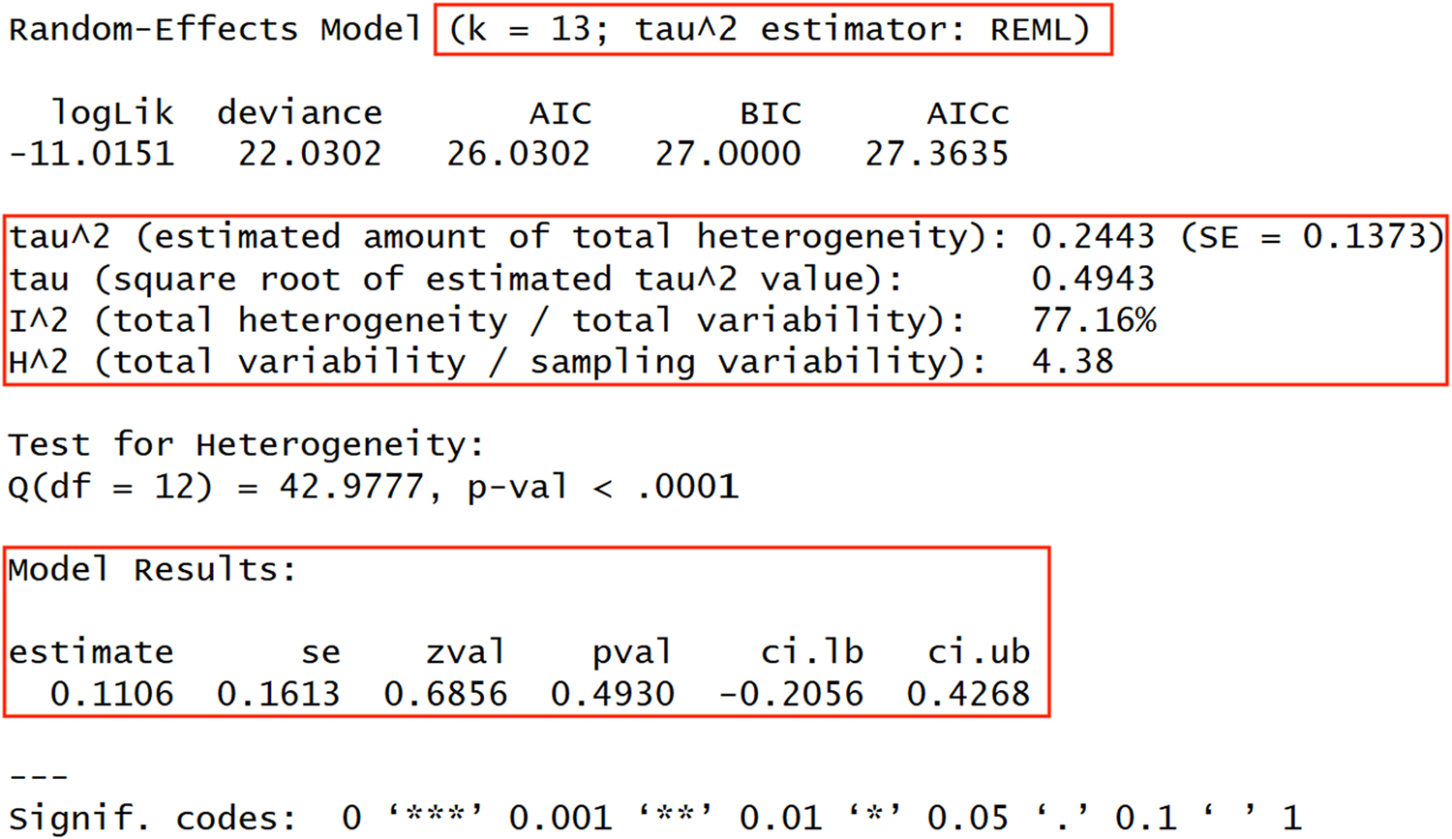
Results of traditional meta-analysis

The second section calculates the overall effect size using the effect sizes (i.e., yi) and sampling variances (i.e., vi). The script is as follows:
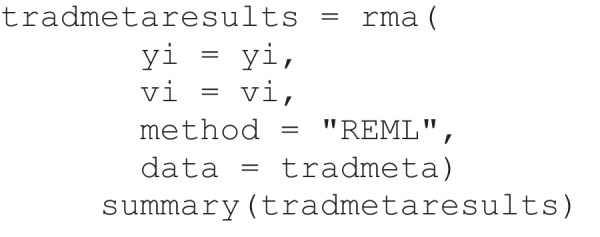


The following output should appear in the console (Fig. 5).

The output contains three important sections to interpret. In the first section, it is important to verify that *k* corresponds to the total number of unique IDs in the data and that the estimator is set to REML. In our analysis, there should be 13 unique IDs. The second section provides the results of the heterogeneity within the meta-analysis. In the example data, the significant *Q* test and ${I}^{2}$ values imply that there is some evidence for heterogeneity in the meta-analysis. The last section provides the overall effect size, with 0.11 representing the Hedges' *g* or overall effect size of the meta-analysis, and *p* >.05 suggesting that the estimate is not statistically significant.

**Fig. 6:**
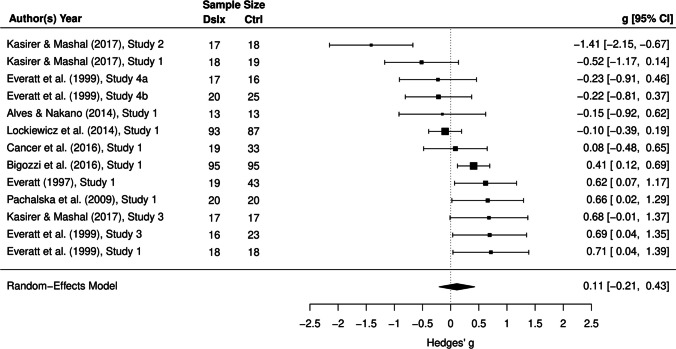
Forest plot of traditional meta-analysis

Additionally, researchers should create a forest plot as it offers an overview of the results of the meta-analysis (Fig. 6) (Dettori et al., 2021). A forest plot should show the effect size of each unique study and the overall meta-analytic effect size (Perera & Heneghan, 2008). The script to create a forest plot and save it as a PDF file is as follows:
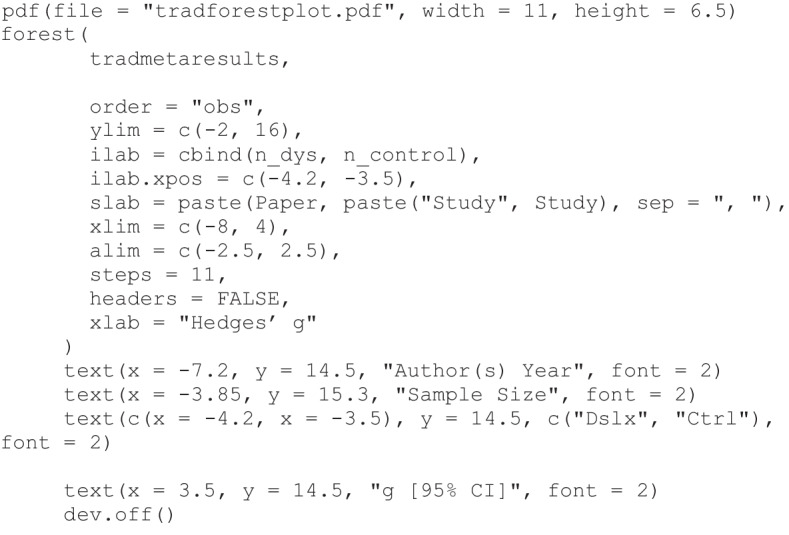

The forest plot in Fig. 6 should consist of three components:

1. The left section listing the study labels;
2. The middle section visually representing the Hedges' *g* of each study;
3. The bottom section displaying the overall meta-analytic effect, and its confidence intervals.

### Checking for publication bias

There are three common methods to check for publication bias: funnel plot (Sterne & Harbord, 2004), rank correlation test (Begg & Mazumdar, 1994), and Egger’s test (Egger et al., 1997). These methods can be respectively executed in R using the *funnel* function, the *ranktest* function, and the *rma* function with the "mods" and "weights" arguments set (refer to *R* code in the current work’s associated OSF for full scripts with annotations).

**Fig. 7:**
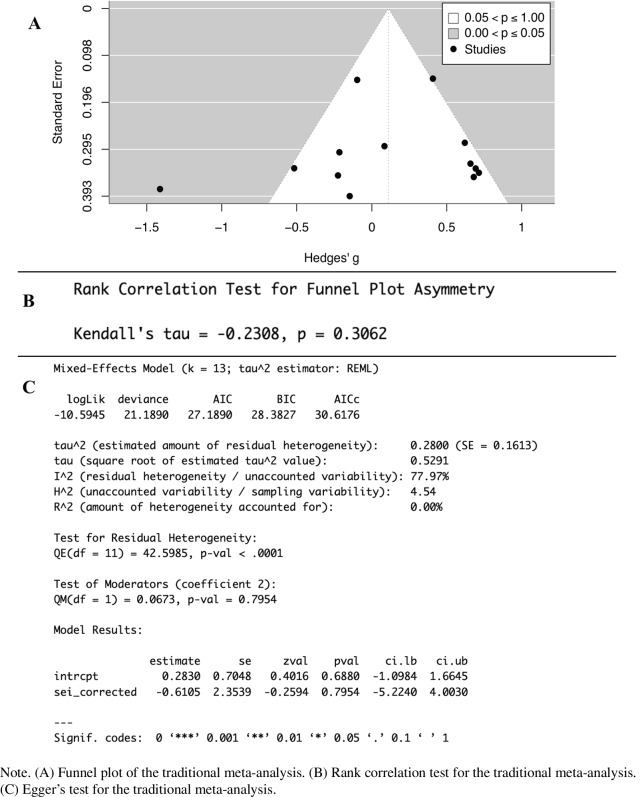
Funnel plot, rank correlation test, and Egger’s test for traditional meta-analysis

Firstly, the interpretation of a funnel plot would depend on the meta-analytic model employed in the analysis. As a random-effects model was employed for this example, the funnel plot would display the relationship between effect sizes on the *x*-axis against its respective standard errors on the *y*-axis (Viechtbauer, 2010⁠). Ultimately, funnel plots are designed to illustrate the relationship between effect sizes (or residuals for mixed-effects models)^7^ and a measure of its precision (Tang & Liu, 2000), which may be its corresponding sampling variances, standard errors, or sample sizes (Viechtbauer, 2010⁠). An asymmetric distribution within the “funnel” shape of the graph (i.e., an uneven spread of dots on both sides of the “funnel”), would indicate publication bias (Fig. 7).

Secondly, a rank correlation test, as a complement to the funnel plot, examines the correlation between the effect size and mean variances of samples (Begg & Mazumdar, 1994). Similar to the funnel plot, a rank correlation test checks the association between effect sizes and the measure of its precision (i.e., standard error), specifically of sampling variances. A non-significant $\tau$, or correlation, suggests a lack of skewness in the funnel plot and no evidence for publication bias (Fig. 7).

Lastly, a modified Egger’s test can be run. Using the original Egger’s test on effect sizes of log-odds ratio and standardized mean difference will result in inflated type I error rates, decreasing the ability to detect publication bias (Macaskill et al., 2001; Pustejovsky & Rodgers, 2019). Hence, a modified measure of precision by Pustejovsky and Rodgers (2019) was proposed and was used to control for the error rates. A non-significant slope estimate would indicate a lack of evidence of publication bias (Fig. 7).

A funnel plot is recommended for visually representing the studies included in the meta-analysis. However, an additional statistical test should also be included as an objective quantification of funnel plot asymmetry (Sterne et al., 2000). The Egger’s test may be utilized as it was shown to be more powerful than the rank correlation test in detecting publication bias (Sterne et al., 2000). However, if the Egger’s test is used in meta-analyses with moderate publication bias and/or fewer than 10 included studies, type I error rate will be inflated (Sterne et al., 2000; Sterne et al., 2011). In such cases, the rank correlation test is recommended instead.

### Moderation analysis

If the moderator is categorical, a subgroup analysis should be conducted. In our example, the categorical moderator was the type of creativity measure. Thus, the *rma* function will be used again, this time with the "subset" argument specifying each level of the moderator (i.e., verbal, non-verbal, and mixed) separately (refer to *R* code in the current work’s associated OSF for full scripts with annotations). 

**Fig. 8:**
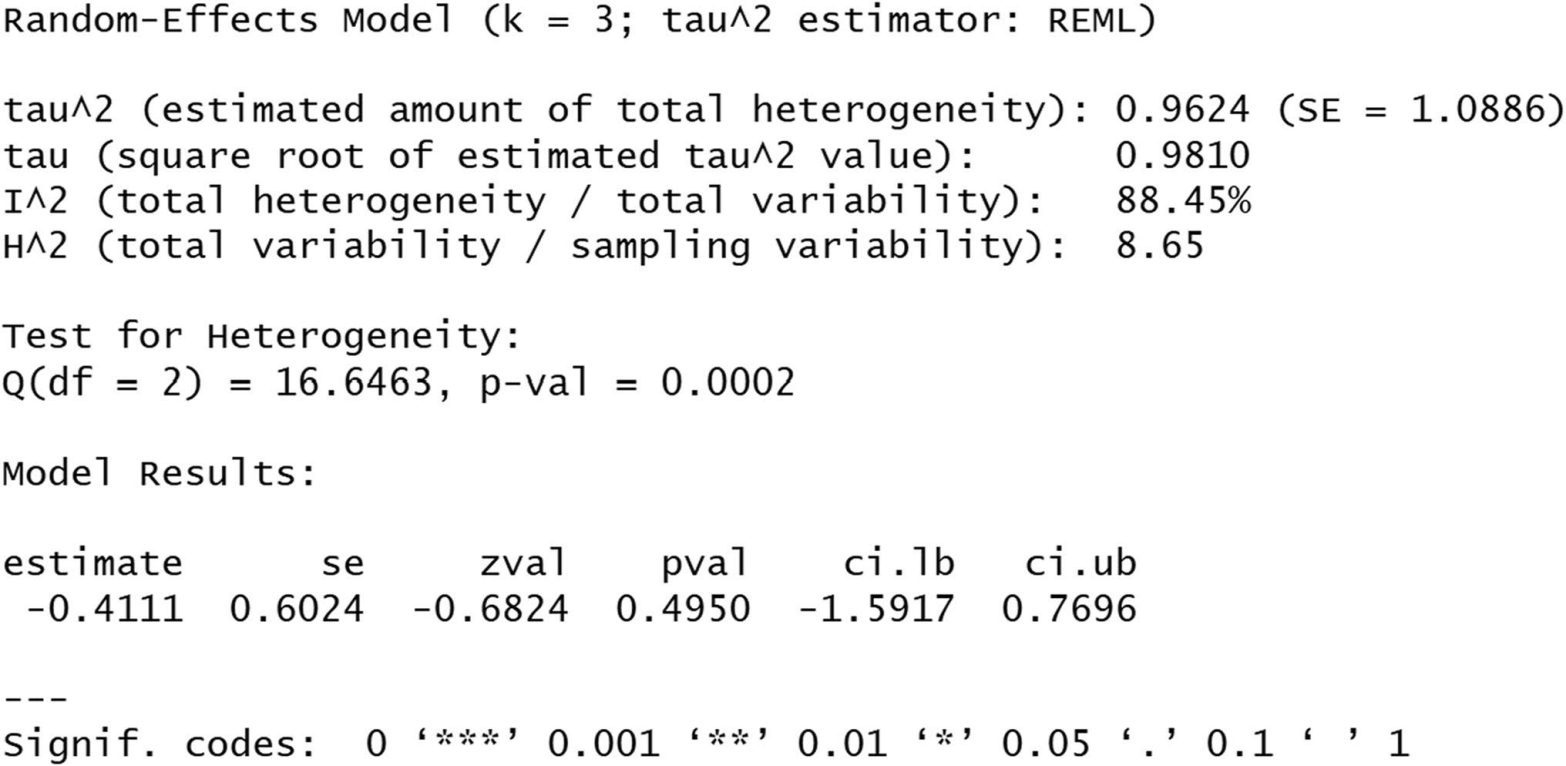
Example output of subgroup analysis for traditional meta-analysis

Figure 8 provides an example of the output for the subgroup analysis. For moderation to exist, the confidence intervals of each type of moderator should not overlap. From the outputs, there is no evidence that the type of creativity measure moderates the effect of dyslexia on creativity as the confidence intervals of each output overlaps.

**Fig. 9:**
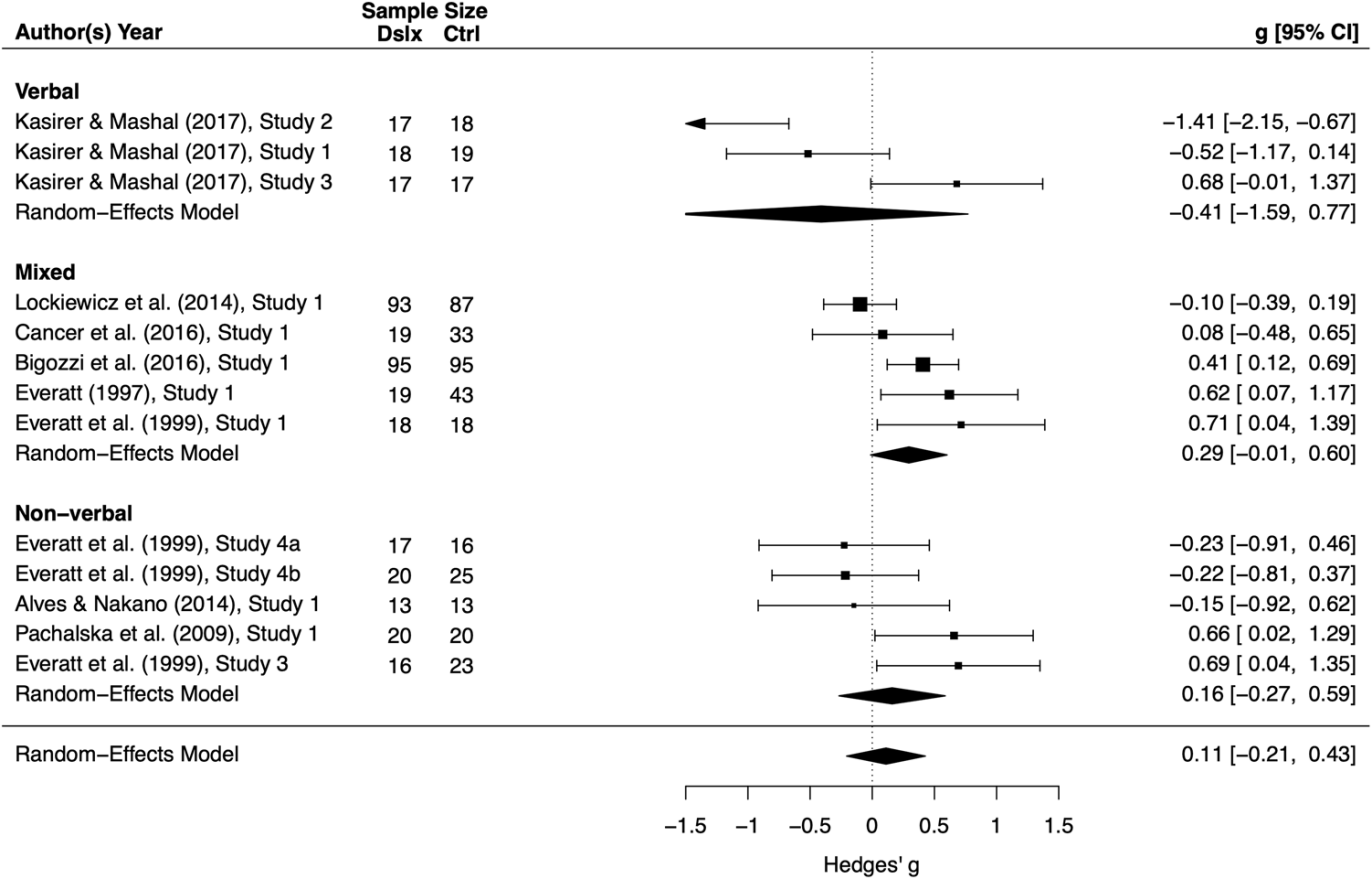
Forest plot categorized by moderators

A forest plot categorized by moderators can be created to visually represent the results of the moderation analysis (Fig. 9). The command to create this forest plot builds upon the previous version, with modifications made to the ordering of the studies. A comprehensive explanation, along with the complete *R* script, can be found in our OSF.

If the moderator was continuous, a meta-regression should be conducted. In our example, the proportion of females was the continuous variable. Thus, the column containing the moderator was included in the argument “mods”. The meta-regression can be executed using the *rma* function once again, this time with the "mods" argument specifying the name of the column containing the values of the continuous moderator (refer to *R* code in the current work’s associated OSF for full scripts with annotations).

**Fig. 10:**
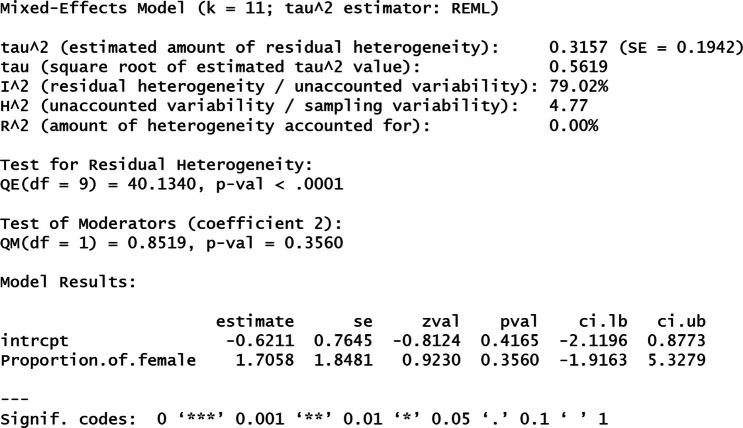
Results of meta-regression for traditional meta-analysis

In the output under model results, the intercept and moderator will be displayed. For moderation to exist, the *p* value of the moderator should be less than .05. From the output of the meta-regression (Fig. 10), there is no evidence that gender has a moderating role on the relationship between dyslexia and creativity.

## Tutorial 3: Multilevel meta-analysis

For this tutorial, we will be referencing a meta-analysis conducted by Hartanto and colleagues (2024) using the research question *“Does the mere presence of smartphones affect cognitive functions*?*”*. A four-level meta-analysis was employed as the included studies violated the assumptions of independence needed for a traditional meta-analysis (for more information, read Hartanto et al., 2024). The complete annotated *R* script for this tutorial is available in the current work’s associated OSF. Additionally, researchers may refer to the *R* Markdown file for a detailed explanation of the *R* script used in this tutorial.

We will demonstrate how applying traditional meta-analytic methods to multilevel data may yield inaccurate or misleading results. Additionally, the multilevel meta-analysis will be conducted with the first level representing each unique study, and the second level representing the lab in which each study was conducted, assuming that each first author was from a different lab.

### Programming

**Fig. 11:**
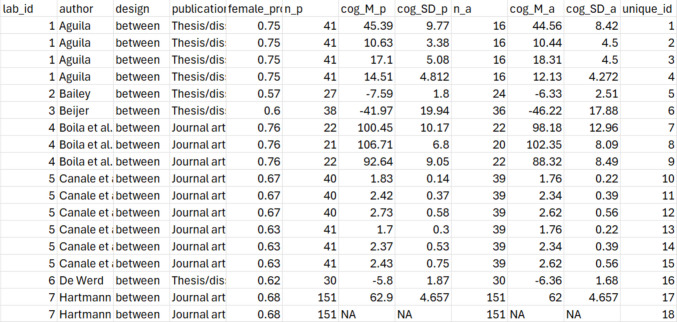
Example of multilevel meta-analysis dataset

For multilevel meta-analysis, the dataset should be in a long format, such that the grouping variable repeats within its column depending on its subset of unique studies (e.g., Fig. 11). If the dataset does not have the unique IDs to identify the papers included in the meta-analysis, these can be generated using the following *R* command: 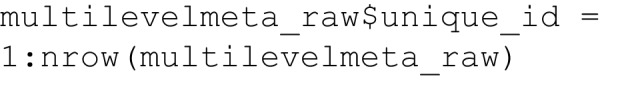

Additionally, some variables may require manual programming. For instance, the current study intends to use labs as the second level of the meta-analysis, but the information was not originally in the dataset. To address this, a new column “lab_id” will be created in the given data set, under the assumption that each author is from a different lab. Fig. 11 provides an example of the recommended dataset for simple and/or multilevel meta-analysis. Table 6 provides a detailed explanation of what each column in the dataset represents.

**Table 6:** Explanation of each column in the dataset

Column	Explanation
lab_id	Describes the grouping variable, which in our example, is the lab that the studies were conducted in
publication	Describes the type of academic work (i.e., journal article, thesis/dissertation, or conference)
female_proportion	Contains the percentage of females in a sample
Starting with n_, cog_M, cog_SD	Represent the sample size, mean, and standard deviation of the dependent variable
Ending with _p, _a	Categorize the treatment and control groups, respectively
unique_id	Contains the unique identifiers for each record. Note that the data used for our example will appear as such only after the aforementioned command, which is during the importing of data

**Fig. 12:**
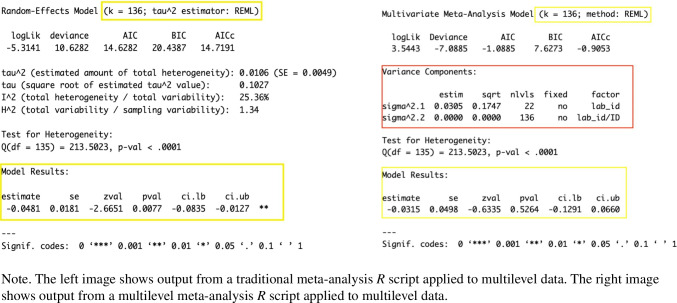
Comparison of traditional and multilevel meta-analysis output using multilevel data

As mentioned earlier, the traditional meta-analysis script will be applied to the data. The results suggest that there is a significant association between smartphone presence and cognitive functions (Fig. 12). However, this output is misleading as the model assumes that effect sizes are independent, despite being applied to data that may be non-independent.

In the output (Fig. 12), *k*, method, and *Q*-statistic should be similar to the output from using the traditional meta-analysis script. Specifically, *k* (i.e., the number of effect sizes) should be 136. The method should remain as REML, and the significant* Q*-statistic implies that there are potential moderators that are not accounted for in the meta-analytic model. However, the new estimate suggests that when including labs as a second level of the meta-analysis (which accounts for the dependence of data), there is a non-significant association between smartphone presence and cognitive functions. Additionally, there is one more section to take note of. Under variance components, the sigma^2.1 estimate represents the level two variance between clusters (i.e., labs) and the sigma^2.2 estimate represents the level one variance within clusters (i.e., unique studies). The output suggests that there is some variability between labs (i.e., estimated value of 0.0305) and that there is no variability within labs (i.e., estimated value of 0.000). nlvls displays the number of groups of each level of the meta-analysis. Accordingly, there were 136 studies within 22 labs.^8^

Therefore, to conduct a multilevel meta-analysis, the script is almost identical except for a key difference. Specifically, the function *rma.mv* will be used instead of *rma* as it takes into account correlated sampling errors and true effects (Viechtbauer, 2010). The function also requires the addition of random effects for the grouping variable. After computing and saving the individual eﬀect sizes into a data frame using the *escalc* function, the overall meta-analytic eﬀect size will be calculated using the *rma.mv* function, with individual eﬀect sizes (i.e., yi) and sampling variances (i.e., vi) specified (refer to the *R* code in the current work’s associated OSF for full scripts with annotations).

### Checking for publication bias

**Fig. 13:**
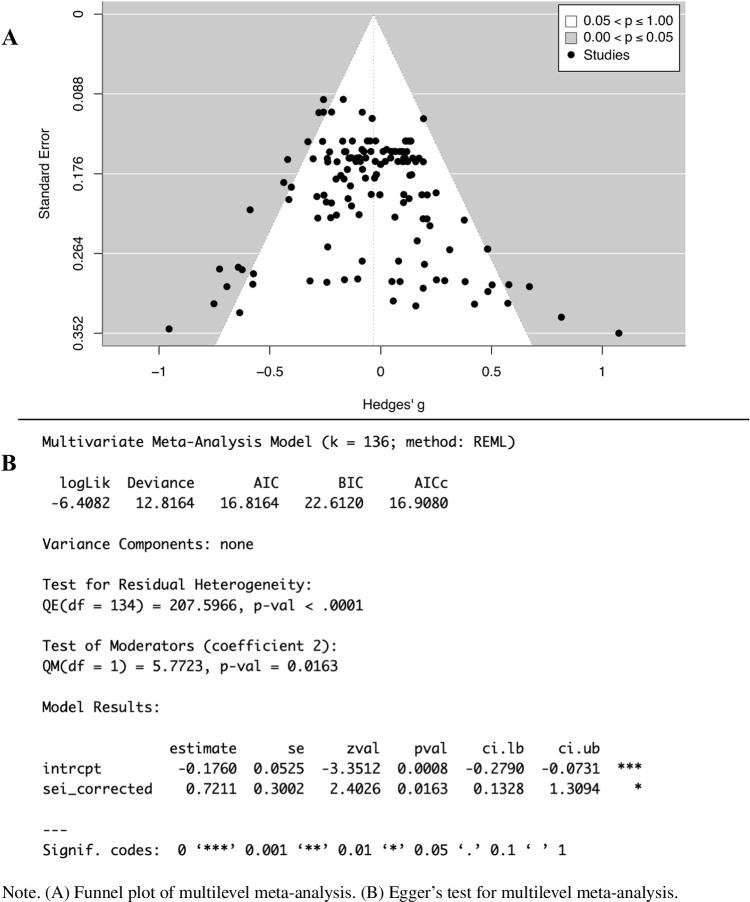
Funnel plot and Egger’s test of multilevel meta-analysis

Lastly, the funnel plot can be created with the same script as before, with the only difference being the data frame. As the funnel plot is symmetric, there is no evidence of publication bias (Fig. 13). However, for multilevel meta-analysis, we do not recommend conducting a rank correlation test as past simulations of the test in a multilevel context resulted in unacceptable ranges of type I error (Fernández-Castilla et al., 2021)—that is, incorrectly rejecting a null hypothesis when it is true. Instead, we recommend the aforementioned modified Egger’s test.

The Egger's test will be run using the *rma.mv* function with changes in the "mods" and "W" arguments (corresponding to the "mods" and "weights" arguments in *rma*). This allows for the Egger's test to account for non-independent data by modeling hierarchical structures (i.e., studies nested within various labs) through additional random effects (for more information, refer to the “Terminologies” section). The significant slope estimate from the output of *rma.mv* implies evidence of publication bias (Fig. 13). 

### Moderation analysis

Approaches to investigate moderation in a multilevel meta-analysis are similar to those of a traditional meta-analysis. For categorical moderators, a subgroup analysis will be conducted, and for continuous moderators, a meta-regression will be conducted. However, the methods for conducting these analyses are slightly different. The *rma.mv* function will be used, with the “subset” argument specifying each level of the moderator (i.e., Journal article, Thesis/dissertation, and Conference) separately (refer to *R* code in the current work’s associated OSF for full scripts with annotations). 

**Fig. 14:**
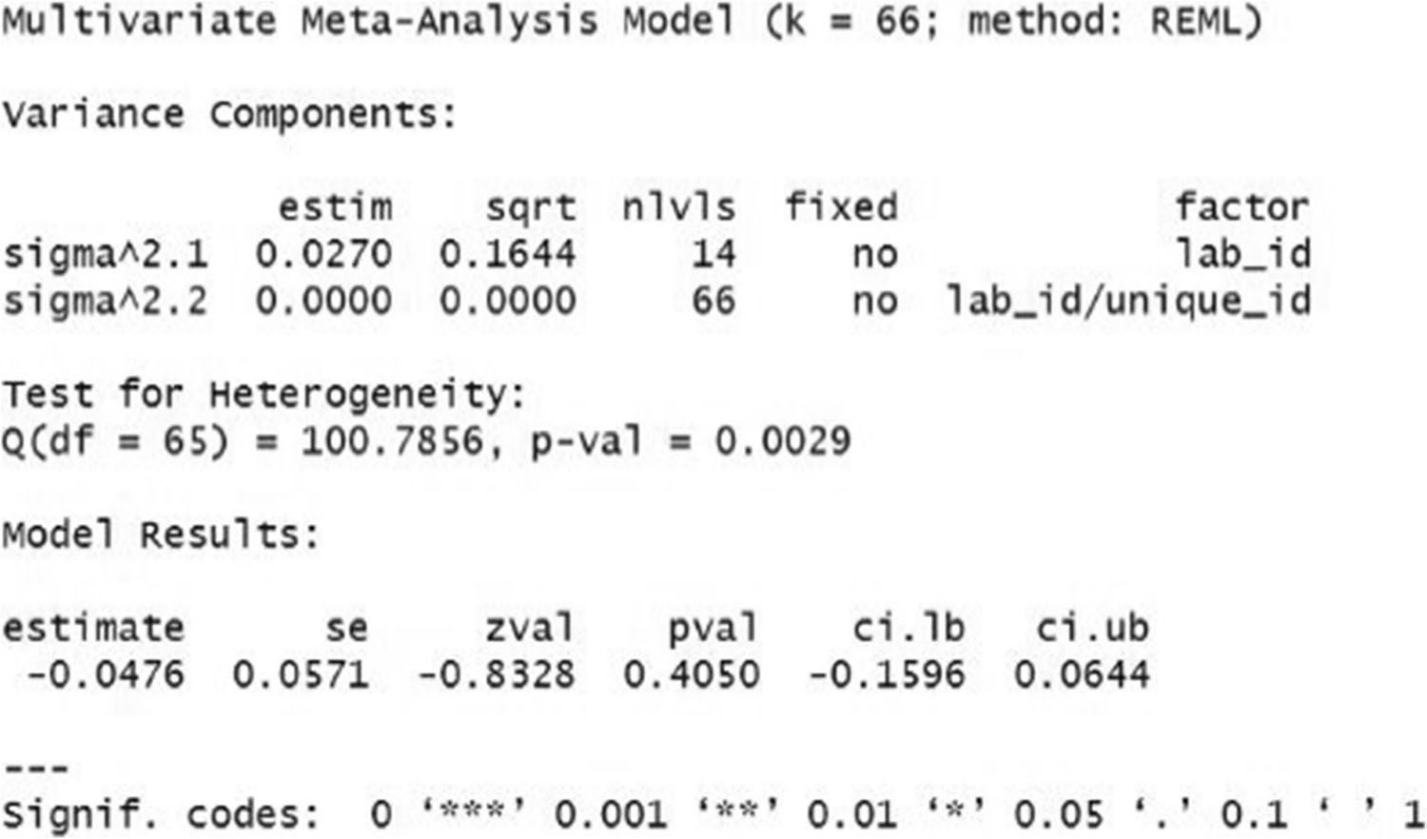
Example output of subgroup analysis for multilevel meta-analysis

Figure 14 provides an example output of the subgroup analysis for a multilevel meta-analysis. As the confidence intervals of each output overlap, we can conclude that there is no evidence that publication type moderates the effect of smartphone presence on cognitive functions.

The meta-regression can be executed using the *rma.mv* function, with the “mods” argument specifying the name of the column containing the values of the continuous moderator (refer to *R* code in the current work’s associated OSF for full scripts with annotations).

**Fig. 15:**
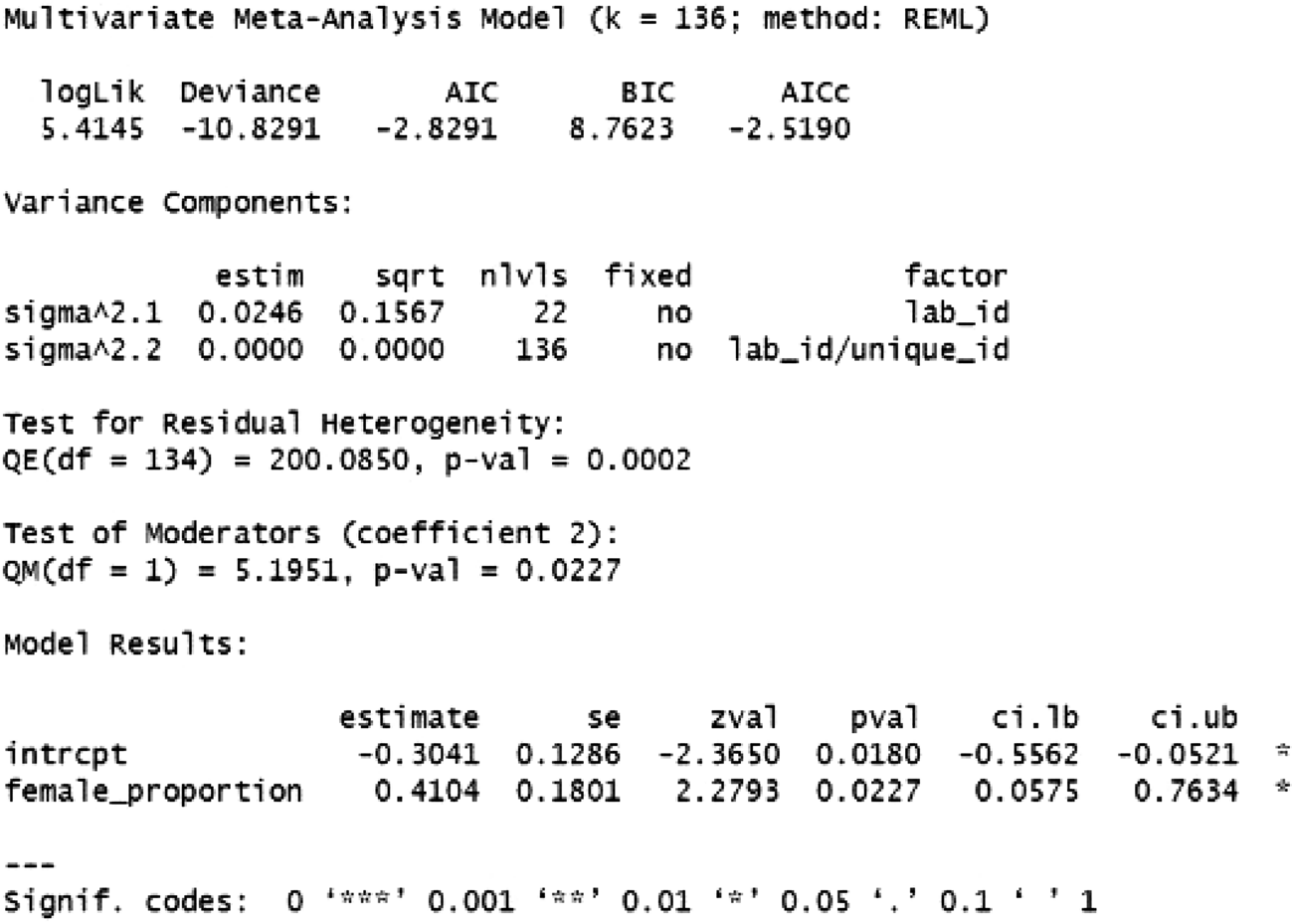
Results of meta-regression for multilevel meta-analysis

According to the output (Fig. 15), we can conclude that there is evidence that gender has a moderating role in the relationship between smartphone presence and cognitive functions.

## Discussion

With the growing volume of studies across research fields, the ability to conduct and evaluate systematic reviews and meta-analyses has become increasingly important. Meta-analyses are especially useful for synthesizing evidence across multiple studies through increased statistical power and aggregating effect sizes (Card, 2012; Kepes et al., 2013). While tutorials on traditional meta-analysis are widely available, many assume prior knowledge, often omitting foundational concepts in research methodology and programming. Existing tutorials also tend to overlook the importance of pre-registering quantitative systematic reviews for research transparency. Furthermore, guidance on alternative meta-analytic methods remains limited, particularly when assumptions of traditional meta-analysis (i.e., independent effect sizes) may be violated (e.g., effect sizes are derived through similar methodologies of the same lab). These alternative approaches include meta-analysis with robust variance estimation 9 and multilevel meta-analysis (Moeyaert et al., 2017). To address these gaps, our tutorial provides a comprehensive step-by-step tutorial for researchers—whether new to evidence synthesis or experienced—on how to conduct systematic reviews, traditional meta-analyses, and multilevel meta-analyses. We hope that this tutorial may serve as a valuable resource for researchers—whether new to evidence synthesis or experienced—seeking to conduct quantitative systematic reviews independently.

### Use of artificial intelligence (AI) tools in the systematic review process

While systematic reviews have traditionally been conducted manually, AI tools have recently become available to automate tasks such as record retrieval, screening, quality appraisal, and data extraction. During the record retrieval stage, tools such as Rayyan can support deduplication of records by automatically identifying and removing duplicate records across databases (Guimarães et al., 2022). In the screening phase, ASReview may be employed to facilitate the process by automating or prioritizing records for reviewer consideration (van de Schoot et al., 2021). When assessing the quality of evidence, RobotReviewer employs natural language processing (NLP)-based models to provide a preliminary appraisal of study quality (Jardim et al., 2022). During the data extraction process, AI tools such as Elicit and ChatPDF can automatically identify and structure relevant data (e.g., sample characteristics, intervention details, and outcomes) from the included records, substantially reducing manual coding (Li et al., 2025; Meliante et al., 2025). In practice, one reviewer may collaborate with an AI tool to conduct data extraction, with a second reviewer cross-checking the extracted data for discrepancies to optimize this laborious process (Helms Andersen et al., 2025).

If AI tools are used at any stage of the systematic review, it is essential that researchers verify AI-generated outputs, as these tools currently lack the critical reasoning and contextual judgement required for nuanced decision-making, and algorithmic biases may influence the validity of the systematic review (Ge et al., 2024; Meliante et al., 2025). Additionally, whenever AI tools are employed at any stage of the systematic review process, researchers should document their use to ensure reproducibility and uphold methodological integrity (Ge et al., 2024; Sampaio, 2025). Overall, while AI tools offer promising ways to enhance the efficiency of the systematic review process, their outputs should be regarded as complementary to, rather than substitutes for, human expertise (Bernard et al., 2025; Ge et al., 2024).

### Further readings

To deepen one’s understanding of systematic reviews and meta-analyses, the following sections provide a selection of recommended readings, organized thematically for ease of reference.

#### Foundations of systematic review and meta-analysis

For researchers seeking more in-depth theoretical explanations of systematic reviews, especially the rationale behind each step of the systematic review process, see Siddaway et al. (2019) and Carlsson et al. (2024). To assess the robustness of the findings of a systematic review, the Grading of Recommendations Assessment, Development, and Evaluation (GRADE; Guyatt et al., 2011) framework may be adopted. For more information on the statistical concepts and analytical techniques involved in a meta-analysis, Harrer et al. (2021) provide a thorough guide. Additionally, Gambarota and Altoè (2024) present the basic concepts of meta-analysis using a simulation-based approach, which may help readers to develop a more intuitive understanding of the underlying statistical processes. Researchers seeking guidance on the procedures for assessing IRR/IRA and the importance of reporting it in systematic reviews or meta-analyses may refer to Lombard et al. (2002).

#### Understanding and converting effect sizes

For a detailed explanation of the concept of effect sizes, researchers may refer to Chapter 6 of Rosenberg et al.’s (2013) *Handbook of Meta-Analysis in Ecology and Evolution* or Chapter 11 of Valentine et al.’s (2019) *The Handbook of Research Synthesis and Meta-Analysis*. Regarding conversion of effect sizes (e.g., conversion of *d* to *r*), researchers may refer to Chapter 7 of Borenstein et al.’s (2021) textbook. For flexible effect size conversion, researchers may utilize *metaConvert*, an *R* package that automatically calculates and converts multiple effect size measures, which Gosling et al. (2025) explain in detail.

#### Addressing publication bias and missing data

Addressing publication bias is another essential component of meta-analytic practice. Fernández-Castilla et al. (2021) provide a detailed overview of various methods, including those discussed in this tutorial (i.e., Egger’s test, rank correlation test, and funnel plots), as well as methods to correct for publication bias, such as the trim-and-fill method. Researchers may also adopt advanced methods to mitigate publication bias, including the use of two frequentist methods—PET-PEESE and selection models—and a Bayesian approach known as robust Bayesian meta-analysis (RoBMA), which integrates both techniques and is thoroughly detailed by Bartoš et al. (2023) using both *R* and JASP software. Alternatively, researchers may use *p*-curve analysis to detect publication bias, as outlined by Simonsohn et al. (2014). In cases where the included studies have missing or partial data, Lajeunesse et al. (2013) suggest statistical methods to replace these missing data with information extracted from the studies.

#### Statistical software and tools

For more information on the other *R* packages available for meta-analysis, Polanin et al. (2017) provide an overview of 63 packages. Additionally, Viechtbauer (2010) offers an in-depth explanation of the *metafor* package used in this tutorial. To evaluate the credibility and reliability of the studies included in the meta-analysis, researchers may utilize the *metameta R* package, which facilitates the straightforward calculation and visualization of study-level statistical power in meta-analyses (Quintana, 2023). Tipton et al. (2019) also review different usages and methods of meta-regression that can be conducted in various programs (e.g., *Stata* and *R*). Additionally, Abbas et al. (2024) introduce a program, Meta-Analysis Accelerator, that can convert different statistics, such as median and interquartile range (IQR), to mean and standard deviation, such that researchers are able to calculate effect sizes from articles that do not provide the standard information required (i.e., mean and standard deviation).

#### Alternative models and advanced meta-analytic designs

For researchers specifically interested in the statistical models used in meta-analysis, Kelley and Kelley (2012) provide a focused discussion on different modelling approaches, such as the maximum likelihood and non-parametric approaches. Stanley et al. (2022) propose an alternative method—unrestricted weighted least squares—for situations where both the variability between studies and the standard errors of their reported effect sizes may share a common underlying cause. Meta-analyses may also vary depending on the research design and complexity of the included studies. Morris and DeShon (2002) provide detailed steps for meta-analyses with studies of various research designs (e.g., between-subjects, within-subjects). Jak and Cheung (2024), Chang and Cheung (2009), and Cheung (2008, 2013, 2015a, b) elaborate on meta-analyses with structural equation modelling, including complex path models such as mediation, in great detail. In addition, Steinmetz and Block (2022) provide recent developments and highlight under-recognized issues in meta-analyses with structural equation modelling. For more advanced or alternative forms of meta-analysis not covered in this tutorial (e.g., individual participant data meta-analysis, meta-analytic Gaussian network aggregation (MAGNA), Bayesian model-averaged meta-analysis), see van Wijk et al. (2023), Epskamp et al. (2022), and Gronau et al. (2021), respectively.

## Supplementary Information

Below is the link to the electronic supplementary material.

Supplementary file1 (PDF 1.07 MB)

## Data Availability

Data and materials for the current work can be downloaded here: https://osf.io/dpqht

## Footnotes

1 Researchers may refer to the online user manual available in the *Publish or Perish* program on how to utilize it.

2 Researchers should note that some databases limit the formats available for downloading the search results. For example, *PubMed* only allows downloads in the .nbib format. As of the time of writing, Zotero allows .nbib files to be imported, while Mendeley does not allow .nbib files to be imported.

3 If the reference manager has a built-in function to remove duplicates, it should be used. However, not all reference managers offer this feature and so, some duplicates may still remain. Therefore, manual checking of duplicates is still recommended.

4 In the identification stage, researchers should report the number of records retrieved from each database and other sources, as well as the number remaining after duplicate removal. The screening stage should outline the number of records included and excluded during both the title/abstract and full-text screening phases, with specific reasons provided for exclusions (e.g., records excluded due to missing data). Finally, the inclusion stage should indicate the number of effect sizes extracted following data extraction.

5 Unlike fixed effects models which assume that all studies estimate a single effect size and any differences in effect sizes are due to sampling error, random-effects models account for true variability between studies.

6 The Mandel–Paule estimation is widely recommended for dichotomous and continuous data (Veroniki et al., 2016) as it is simple to implement and less biased than the DerSimonian and Laird method when the heterogeneity is moderate to high (Langan et al., 2017).

7 However, funnel plots are usually made from random- or fixed-effects models. For mixed-effects models, the funnel plot would display the residuals on the *x*-axis against its respective standard errors on the *y*-axis (Viechtbauer, 2010⁠).

8 Given the large number of records, a forest plot of all studies may not be appropriate as it may appear to be cluttered and difficult to interpret.

9 Multilevel meta-analysis is preferred to meta-analysis with robust variance estimation as multilevel meta-analysis allows for the examination of both between-study variance and within-study variance, while robust variance estimation meta-analysis only allows for the examination of between-study variance.

## Code availability

Code for the current work is available here: https://osf.io/dpqht
