# Supplementary material for: From review to synthesis: A step-by-step methodological guide to systematic reviews and multilevel meta-analyses
Source: Behav Res Methods. 2026 Jun 16;58(7):197. doi: 10.3758/s13428-026-02961-x (PMC13272617; doi:10.3758/s13428-026-02961-x)
Supplement: Supplementary file 1 — Supplementary file1 (PDF 1.07 MB) [file 13428_2026_2961_MOESM1_ESM.pdf]

Table of Contents

S1. Examples of Papers Utilising Systematic Reviews and Meta-Analyses 2

S2. FINER Criteria 4

S3. Documenting the Retrieval Process 8

S4. Data Import and Preparation in RStudio 9

S5. Inter-Rater Agreement Methods 15

Cohen's κ 15

Percent Agreement Method for Calculating IRR/IRA 16

S6. Data Structure Formats 20

S1. Examples of Papers Utilising Systematic Reviews and Meta-Analyses

Table S1.1

Published Examples of Meta-Analytic Research Questions Across Psychology

Authors	Research Questions	Number of Records Included	Years of Included Records (Inclusive)	Pre-Registered?	Type of Meta-Analysis	Model
Bolier et al. (2013)	What are the effects of specific positive psychology interventions in the general public and in people with specific psychosocial problems?	40	1980–2012	No	Traditional	Random-effects
Chen et al. (2024)	How does human-animal interaction influence human prosociality, and what are the potential moderators of this relationship?	20	1999–2021	No	Multilevel	Random-effects
Hall et al. (2016)	What are the effects of culturally-adapted psychological interventions, as compared to not having an intervention or other types of intervention, on psychopathology outcomes? What are the moderators influencing this effect?	78	1992–2015	No	Multilevel	Random-effects
Hartanto et al. (2024)	What is the effect of the presence of smartphones on cognitive functions?	29	2017–2022	No	Multilevel	Random-effects
Hoogsteder et al. (2023)	What is the effectiveness of mindfulness-based interventions on externalising problem behaviour? Does the involvement of parents or type of treatment affect the effectiveness?	14	2008–2021	No	Multilevel	Random-effects

Huang (2022)	What is the relationship between problematic social media use and mental health?	123	2009–2020	No	Traditional	Random-effects
Jiang et al. (2013)	What is the association between prostatitis and prostate cancer?	20	1994–2012	No	Traditional	Random- and Fixed-effects
Kasturiratna et al. (2025)	How does digital mental health interventions affect attention-deficit hyperactivity disorder?	23	2011-2024	Yes	Multilevel	Random-effects
Katebi et al. (2021)	What is the association between job satisfaction and job performance?	113	2012-2019	No	Traditional	Random- and Fixed-effects
Lua et al. (2023)	What is the overall relationship between the need for cognition and well-being?	50	1986–2021	No	Multilevel	Random-effects
Majeed et al. (2023)	How do anxiety disorders affect executive functions, specifically in terms of reaction time and accuracy?	55	1990–2019	No	Multilevel	Random-effects
Majeed et al. (2021)	What is the relationship between clinically diagnosed dyslexia and creativity?	9	1997–2017	No	Traditional	Random-effects
O’hara & Swain (1996)	What are the rates and risk factors of postpartum depression?	77	1979-1995	No	Traditional	Fixed-effects
Ogilvie et al. (2011)	What is the relationship between executive functioning and antisocial behaviour? What are the possible moderators affecting this association?	126	1942–2010	No	Traditional	Random-effects

Ong et al. (2020)	What is the relationship between positive affect and pain severity among adults with chronic non-cancer pain?	29	1981–2018	Yes	Traditional	Random-effects
Wong et al. (2022)	What is the relationship between social media and well-being, in the context of COVID-19?	38	2020–2021	No	Multilevel	Random-effects

S2. FINER Criteria

A helpful framework for crafting research questions would be the FINER criteria: Feasible, Interesting, Novel, Ethical, and Relevant (Fandino, 2019; Willis, 2023). Table S2.1 provides an overview of the FINER criteria and illustrates how each criterion can provide a structured basis for researchers to justify and proceed confidently with a quantitative systematic review on the topic, using the example question that aims to investigate the association between clinically diagnosed dyslexia and creativity.

Table 2.1

Overview of FINER Criteria in the Context of a Quantitative Systematic Review

Component	Definition	Assessment Questions	Example
Feasibility	Ensures that the research question can be addressed through a quantitative systematic review	Are there at least five empirical studies available to provide data for a quantitative systematic review?	At least 5 empirical studies are available to conduct a quantitative systematic review on the association between clinically diagnosed dyslexia and creativity
Interest	Ensures that the research question will capture the attention and curiosity of a broad audience	Are people intrigued by or curious about the research question?	Multiple empirical studies have been conducted to study the relationship between clinically diagnosed dyslexia and creativity
Novel	Ensures that the research question identifies inconsistencies within a topic and explores potential ways to establish consensus in the current literature	Will the findings of the quantitative systematic review contribute new knowledge to the existing literature?	A quantitative systematic review on the relationship between clinically diagnosed dyslexia and creativity may resolve the discrepancies in the current research
Ethical	Ensures that the review adheres to ethical research practices, even though it may not involve human subjects directly	Are ethical research practices adhered to in the review (e.g., no plagiarism, transparent in its methodology)?	The quantitative systematic review adhered to ethical research practices, such as transparency in its methodology, appropriate citation of sources, and avoidance of plagiarism.
Relevant	Ensures that the impact of the research question would be significant in policy-making and/or advances the theoretical understanding about the psychological concepts and theories	Will the findings of the quantitative systematic review inform decision-making processes and/or provide meaningful theoretical understanding about the psychological concepts and theories?	The findings of the quantitative systematic review could provide insights into possible moderators (e.g., age, type of creativity, severity of dyslexia) that influence the relationship between clinically diagnosed dyslexia and creativity, and inform future research directions, policy decisions, and/or theoretical models

S3. Documenting the Retrieval Process

Table S3.1

Template for Documenting Records

**Database Search**<br><br>*Keywords: [Insert the Search String]*			
**Database**	**Number of Studies Retrieved**	**Date of Retrieval**	**Retrieval Conducted by Who**
ERIC			
PsycInfo			
PubMed			
Scopus			
Web of Science			
**TOTAL FROM DATABASES**<br><br>**With duplicates**			
**TOTAL FROM DATABASES**<br><br>**Without duplicates**			

S4. Data Import and Preparation in RStudio

Before analysing the data, the dataset must first be imported into RStudio. There are a few terminologies that researchers need to be familiar with when navigating the interface of RStudio, which would be: the console, the environment, and the editor. First, the console is where the results and output of the code will appear. Next, the environment is where loaded datasets, variables, and objects are listed. Lastly, the editor is the space for researchers to write their code. Figure S4.1 illustrates the interface of RStudio.

Figure S4.1

Interface of RStudio

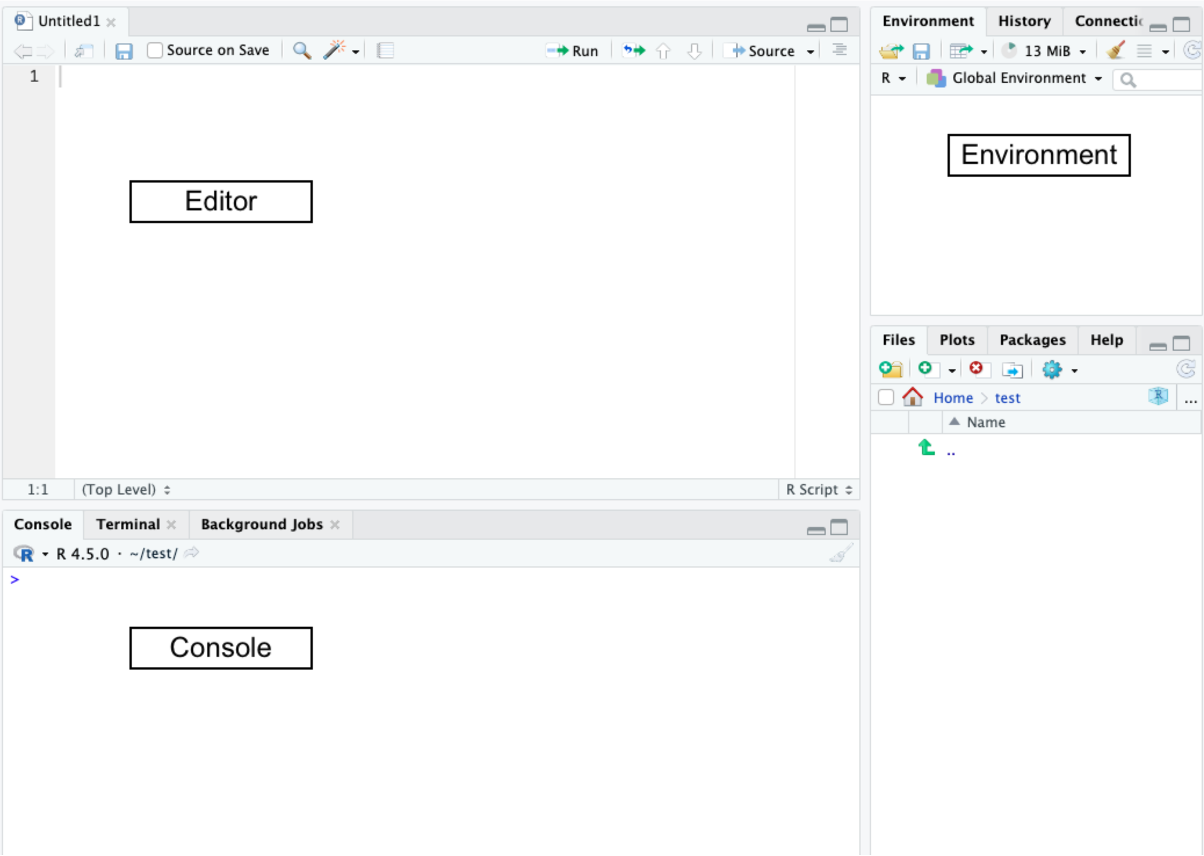

To begin, researchers should open a new R Script to write their code. This can be done using either one of the two proposed methods. First, in the bottom-right panel of the RStudio interface, click on “New Blank File” and select “R Script” from the dropdown menu (Figure S4.2). Alternatively, researchers may navigate to the top menu bar, click on “File” and select “New File” from the dropdown menu, and click on “R Script” (Figure S4.3).

Figure S4.2

Opening R Script

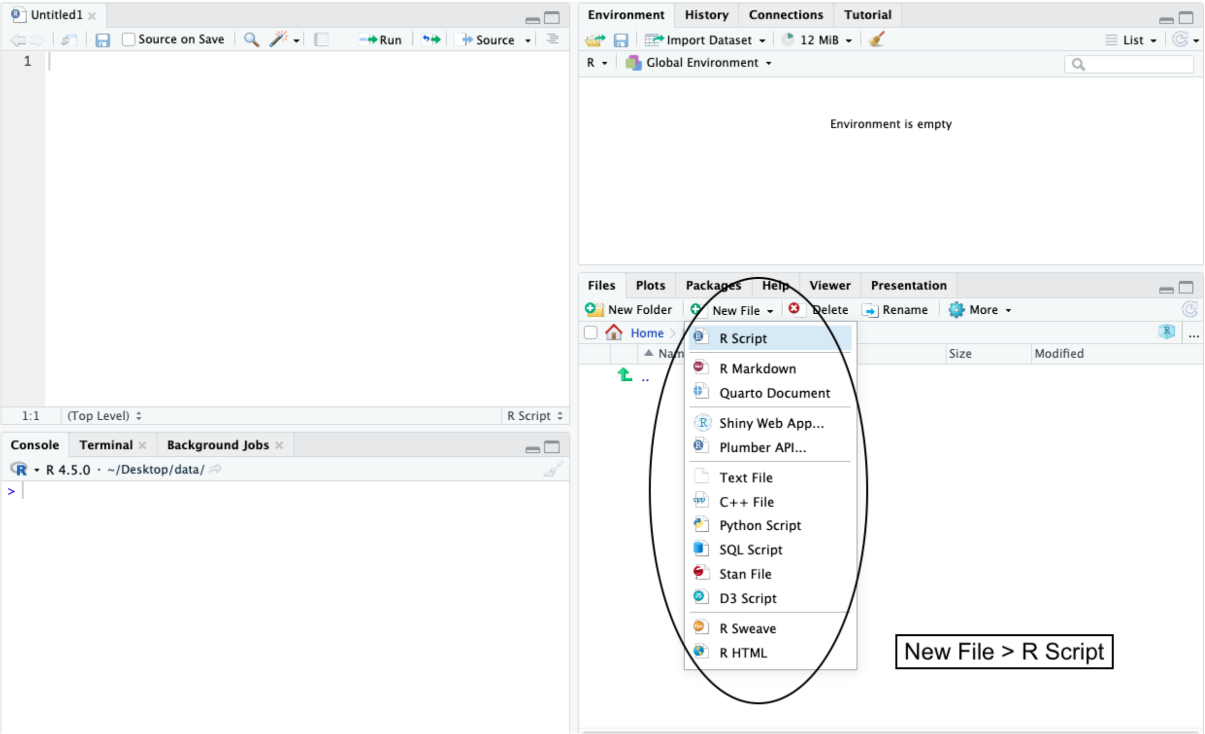

Figure S4.3

Opening R Script

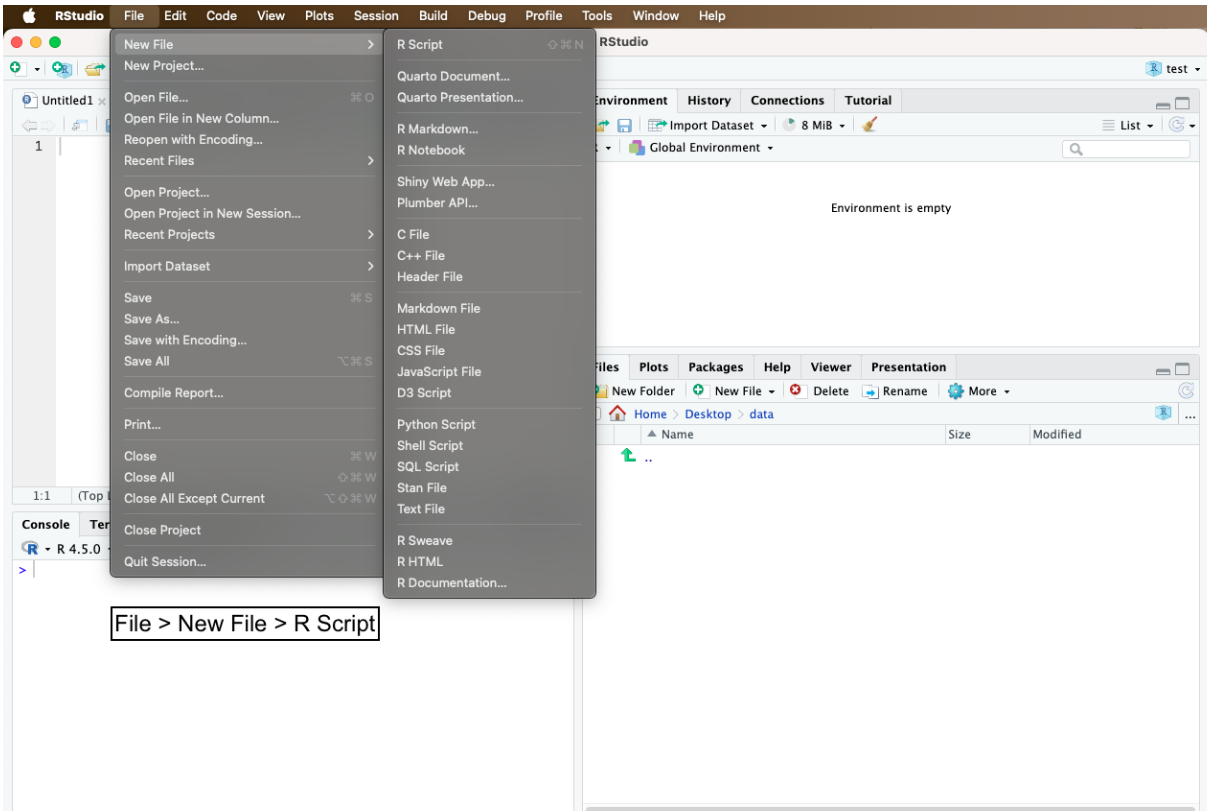

Once the *R* Script is open, the next step is to set the working directory. Setting the working directory ensures that RStudio knows where to locate the dataset that is to be analysed. This can be achieved through one of the two proposed ways. Researchers may navigate to the bottom-right panel of the RStudio and look for the folder where the dataset was saved. Click on the cogwheel labelled “More” and select “Set as Working Directory” from the dropdown menu (Figure S4.4). Alternatively, researchers may navigate from their menu bar, click on “Session”, followed by “Set as Working Directory”, and select “Choose Directory” from the dropdown menu (Figure S4.5). Afterwards, select the folder where the data file was saved and click open. For both methods, a command starting with `setwd()`

should appear in the console (Figure S4.6). Researchers are encouraged to copy that command and paste it into the R Script editor for future reference (Figure S4.6). Once the working directory is set, researchers may use the following code to read in the data:

```
data = read.csv("SPC.csv")
```

In this command, data serves as the object where the dataset is saved to. Researchers may replace "data" with a name of their choice. Researchers can also replace `"SPC.csv"` with the actual name of their dataset file.

Figure S4.4

Setting the Working Directory

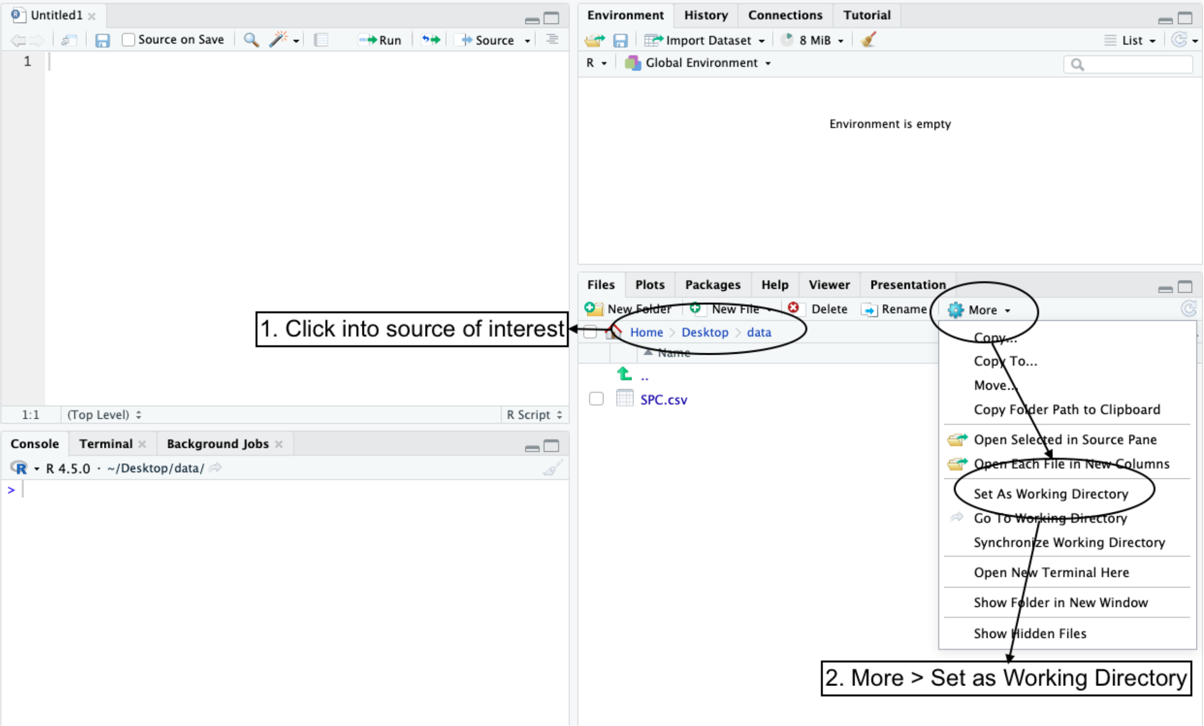

Figure S4.5

Setting the Working Directory

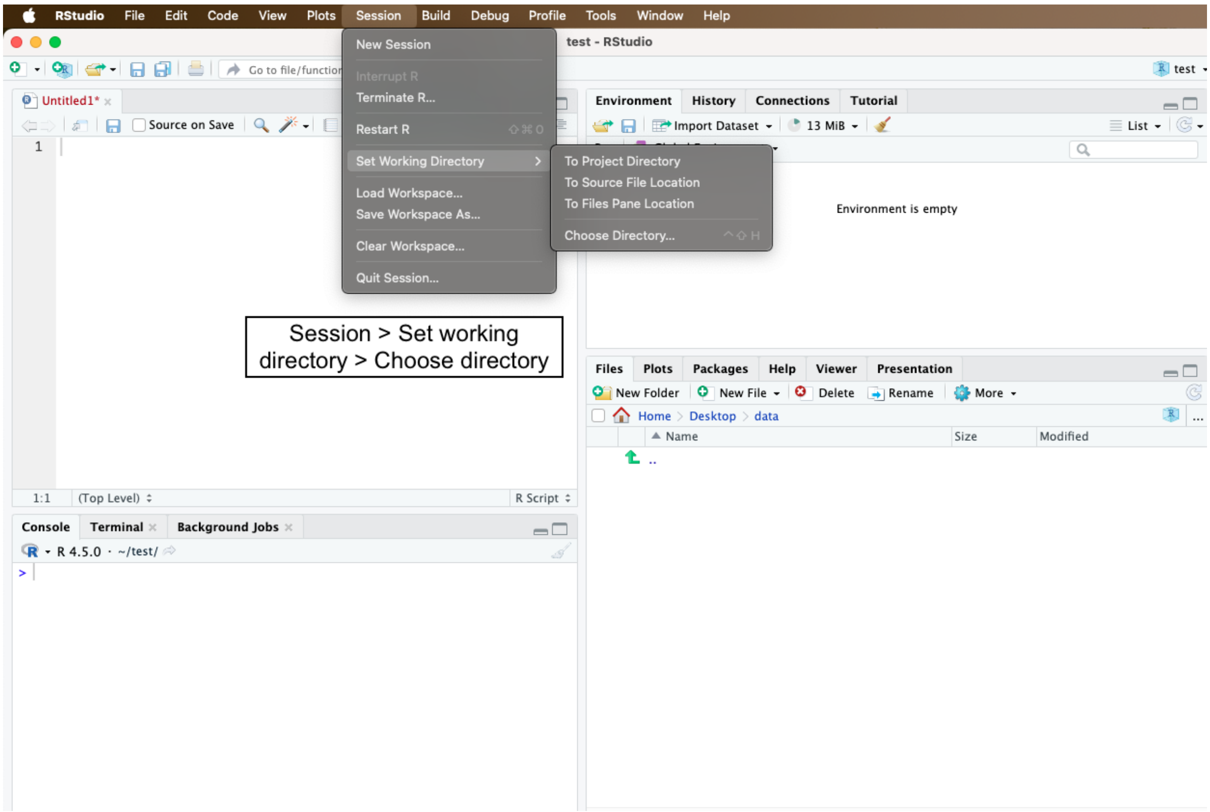

Figure S4.6

Saving Working Directory Code into the Console

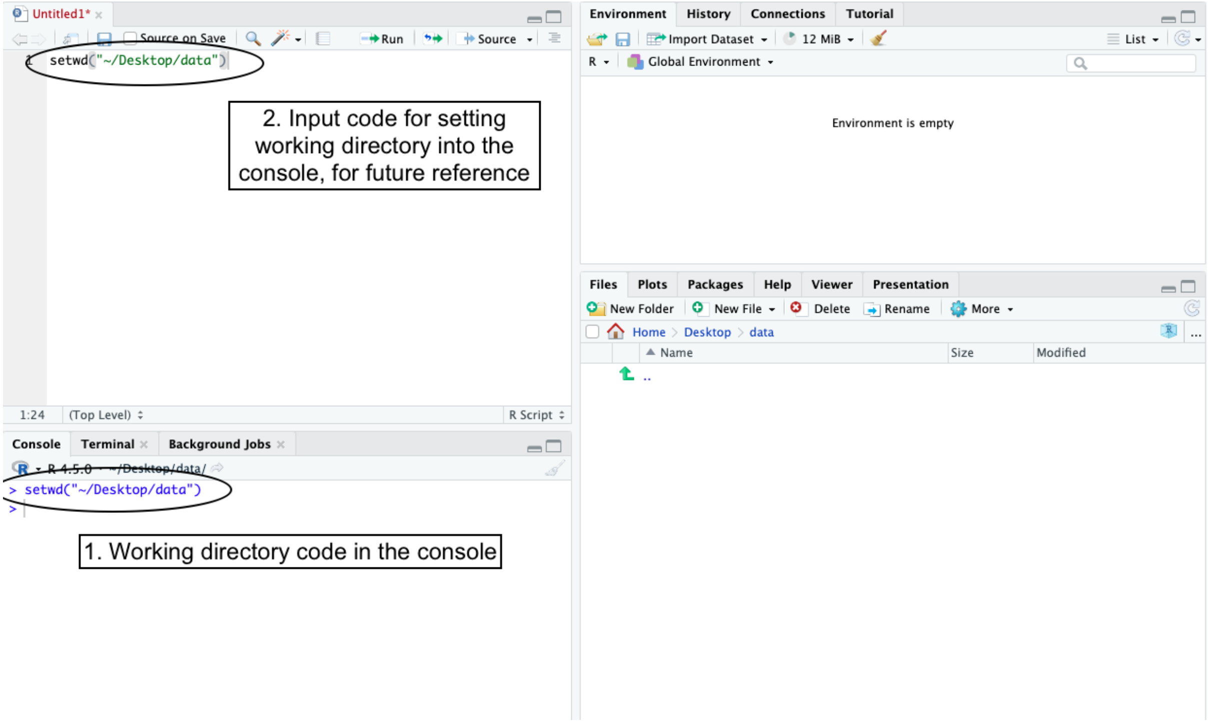

S5. Inter-Rater Agreement Methods

Cohen's κ

Cohen's κ, symbolised by the lowercase Greek letter, κ, ranges from 0 to +1. A value of 0 indicates the expected agreement between the screeners by random chance, while +1 represents perfect agreement between the screeners (McHugh, 2012). Calculating Cohen's κ is another method researchers may employ in calculating IRR/IRA in this stage. The following contingency table illustrates how researchers may compute Cohen's Kappa:

	Rater B: Yes	Rater B: No	Total
Rater A: Yes	*a* (e.g., 3)	*c* (e.g., 5)	*e* (e.g., 8)
Rater A: No	*b* (e.g., 1)	*d* (e.g., 7)	*f* (e.g., 8)
Total	*g* (e.g., 4)	*h* (e.g., 12)	*N* (e.g., 16)

1. To calculate *Po*, which is the proportion of cases where the screeners agree:

$$Po = \frac{a + d}{N}$$

Substituting values:

$$Po = \frac{3 + 7}{16} = 0.625$$

2. To calculate *Pe*, which is calculated based on the marginal totals of the contingency table:

$$Pe = [(\frac{e}{N}) \times (\frac{g}{N})] + [(\frac{f}{N}) \times (\frac{h}{N})]$$

Substituting values:

$$Pe = [(\frac{8}{16}) \times (\frac{4}{16})] + [(\frac{8}{16}) \times (\frac{12}{16})] = 0.5$$

3. To calculate Cohen's Kappa,

$$\kappa = \frac{Po - Pe}{1 - Pe}$$

Substituting values:

$$\kappa = \frac{0.625 - 0.5}{1 - 0.5} = 0.25$$

To interpret the final results of the IRR/IRA, Table S5.1 consolidates the standard interpretation of Cohen's κ value (Bajpai et al., 2015; Landis & Koch, 1977; McHugh, 2012). Higher values indicate greater agreement between the raters. Researchers should note that agreement levels below 'moderate' may require retraining the screeners or revising the screening criteria (McHugh, 2012).

Table S5.1

Interpretation of the Values of Cohen's κ

**Value of Cohen's κ**	**Level of Agreement**
.00-.20	None
.21-.39	Minimal
.40-.59	Weak
.60-.79	Moderate
.80-.90	Strong
Above .90	Almost perfect

Percent Agreement Method for Calculating IRR/IRA

Each screener will provide their evaluations for each criterion per paper by indicating their agreement or disagreement. To determine the overall inter-rater reliability, the percent agreement values for all the criteria are averaged, providing a raw agreement rate expressed as a percentage (%). The formula for this method is as follows:

$$\text{Raw Agreement} = \frac{Number\ of\ agreements}{Total\ number\ of\ records\ rated} \text{ x } 100$$

Each screener will provide their evaluations in a singular spreadsheet. For each criterion in each paper, screeners will indicate their agreement or disagreement as suggested:

- 'TRUE' or 'MATCH' or '1' to indicate agreement between the screeners
- 'FALSE' or 'RESOLVE' or '0' to indicate disagreement between the screeners

This process should be completed for each criterion across all papers. Afterwards, the inter-rater reliability for each criterion can be calculated using the aforementioned formula. This calculation should be repeated for each criterion individually. For example, if 30 out of 40 evaluations for a criterion are in agreement, the IRR/IRA for that criterion will be calculated as so: $\frac{30}{40} \times 100\% = 75\%$.

To determine the overall inter-rater reliability, the percent agreement values for all the criteria are averaged, providing a raw agreement rate expressed as a percentage (%). To facilitate the process, we recommend using the following formulas that can be used in either Google Sheets or Microsoft Excel, depending on which software the researcher has chosen to conduct the abstract and title screening with:

- =IF formula: To automate the marking of agreement or disagreement between the screeners

- =COUNTIF and =AVERAGE formula: To calculate the raw agreement rate between the screeners

Table S5.2 illustrates the process of calculating IRR or IRA with the percent agreement method.

Table S5.2

Percent Agreement Method Process of Calculating Inter-Rater Reliability with Microsoft Excel or Google Sheets

Screener 1 Abstract Screening Sheet Tab

	Column A	Column B	Column C	Column D
Row E	**Paper**	**Criteria 1**	**Criteria 2**	**Criteria 3**
Row F	ABC	Yes	Yes	No
Row G	EFG	No	Yes	No
Row H	HIJ	Yes	Yes	Yes

Screener 2 Abstract Screening Sheet Tab

	Column A	Column B	Column C	Column D
Row E	**Paper**	**Criteria 1**	**Criteria 2**	**Criteria 3**
Row F	ABC	Yes	No	Yes
Row G	EFG	No	Yes	Yes
Row H	HIJ	Yes	Yes	Yes

Inter-Rater Calculation (1)

	Column A	Column B	Column C	Column D
Row E	**Paper**	**Criteria 1**	**Criteria 2**	**Criteria 3**
Row F	ABC	=if(BF of Screener 1 = BF of Screener 2, “MATCH”, “RESOLVE”)	=if(CF of Screener 1 = CF of Screener 2, “MATCH”, “RESOLVE”)	=if(DF of Screener 1 = DF of Screener 2, “MATCH”, “RESOLVE”)
Row G	EFG	=if(BG of Screener 1 = BG of Screener 2, “MATCH”, “RESOLVE”)	=if(CG of Screener 1 = CG of Screener 2, “MATCH”, “RESOLVE”)	=if(DG of Screener 1 = DG of Screener 2, “MATCH”, “RESOLVE”)
Row H	HIJ	=if(BH of Screener 1 = BH of Screener 2, “MATCH”, “RESOLVE”)	=if(CH of Screener 1 = CH of Screener 2, “MATCH”, “RESOLVE”)	=if(DH of Screener 1 = DH of Screener 2, “MATCH”, “RESOLVE”)

Inter-Rater Calculation (2)

	Column A	Column B	Column C	Column D
Row E	**Paper**	**Criteria 1**	**Criteria 2**	**Criteria 3**
Row F	ABC	MATCH	RESOLVE	RESOLVE
Row G	EFG	MATCH	MATCH	RESOLVE
Row H	HIJ	MATCH	MATCH	MATCH
Row I		=COUNTIF(BF :BH, “MATCH”) /3	=COUNTIF(CF :CH, “MATCH”) /3	=COUNTIF(DF :DH, “MATCH”) /3
= AVERAGE (BI:DI) %				

The percent agreement method is straightforward to calculate (Gisev et al., 2013), with the results that are easy to interpret (Bajpai et al., 2015). However, the percent agreement method does not account for chance agreement, potentially overestimating the

agreement rate (Bajpai et al., 2015; McHugh, 2012). In contrast, Cohen's κ accounts for the possibility of guessing, but yields results that are less intuitive to interpret (Bajpai et al., 2015). The choice of method depends on the likelihood of the screeners guessing their evaluation of each criterion. If guessing is of a significant concern, Cohen's κ would be more appropriate for calculating IRR/IRA (McHugh, 2012). On the other hand, if the screeners are well-trained and guessing is unlikely, the percent agreement method may be sufficient (McHugh, 2012).

S6. Data Structure Formats

There are two main formats: wide and long. In a wide format, each study will occupy a single row, with separate columns representing a variable or outcome of interest, making it suitable for meta-analyses where the included studies report the same set of variables In a long format, each study may have multiple rows, with each row corresponding to a variable or outcome of interest, which is ideal for meta-analyses with multiple outcomes or for more complex methods such as multilevel meta-analyses. The format of the dataset also depends on the *R* packages that will be utilised. For example, visualisation packages such as *ggplot2* require a long format while both forms of datasets are suitable to be used with the meta package. Ultimately, the format of the dataset depends on two key factors: the type of meta-analysis being conducted and the requirements of the *R* packages. Figures S6.1 and S6.2 illustrate examples of wide and long formats respectively.

Table S6.1

Example of a Wide Format Dataset

Author	Effect Size Outcome 1	Effect Size Outcome 2
ABC	0.4	0.5
EFG	0.1	0.3
HIJ	0.5	0.5

Table S6.2

Example of a Long Format Dataset

Author	Variable of Interest	Effect Size
ABC	Outcome 1	0.1
ABC	Outcome 2	0.4
EFG	Outcome 1	0.3

References

Bajpai, S., Bajpai, R., & Chaturvedi, H. (2015). Evaluation of inter-rater agreement and inter-rater reliability for observational data: An overview of concepts and methods. *Journal of the Indian Academy of Applied Psychology*, *41*, 20–27.

Bolier, L., Haverman, M., Westerhof, G. J., Riper, H., Smit, F., & Bohlmeijer, E. (2013). Positive psychology interventions: A meta-analysis of randomized controlled studies. *BMC Public Health*, *13*(1), 119. https://doi.org/10.1186/1471-2458-13-119

Chen, N. R. Y., Majeed, N. M., Lai, G. J., Koh, P. S., Kasturiratna, K. T. A. S., Kaur, M., Ho, A. Z. Y., Yong, J. C., & Hartanto, A. (2024). Human–animal interaction and human prosociality: A meta-analytic review of experimental and correlational studies. *Anthrozoös*. https://doi.org/10.1080/08927936.2023.2288745

Fandino, W. (2019). Formulating a good research question: Pearls and pitfalls. *Indian Journal of Anaesthesia*, *63*(8), 611. https://doi.org/10.4103/ija.IJA_198_19

Gisev, N., Bell, J. S., & Chen, T. F. (2013). Interrater agreement and interrater reliability: Key concepts, approaches, and applications. *Research in Social and Administrative Pharmacy*, *9*(3), 330–338. https://doi.org/10.1016/j.sapharm.2012.04.004

Hall, G. C. N., Ibaraki, A. Y., Huang, E. R., Marti, C. N., & Stice, E. (2016). A meta-analysis of cultural adaptations of psychological interventions. *Behavior Therapy*, *47*(6), 993–1014. https://doi.org/10.1016/j.beth.2016.09.005

Hartanto, A., Lua, V. Y. Q., Kasturiratna, K. T. A. S., Koh, P. S., Tng, G. Y. Q., Kaur, M., Quek, F. Y. X., Chia, J. L., & Majeed, N. M. (2024). The effect of mere presence of smartphone on cognitive functions: A four-level meta-analysis. *Technology, Mind, and Behavior*, *5*(1: Spring 2024). https://doi.org/10.1037/tmb0000123

Hoogsteder, L. M., Van Os, R. C. J., Lutjens, J. B., Sweers, N., & Stams, G. J. M. M. (2023).

A multilevel meta-analysis on the effect of mindfulness-based interventions in reducing externalizing problem behavior in adolescents. *International Journal of Stress Management*, *30*(3), 309–320. https://doi.org/10.1037/str0000285

Huang, C. (2022). A meta-analysis of the problematic social media use and mental health. *The International Journal of Social Psychiatry*, *68*(1), 12–33. https://doi.org/10.1177/0020764020978434

Jiang, J., Li, J., Yunxia, Z., Zhu, H., Liu, J., & Pumill, C. (2013). The role of prostatitis in prostate cancer: Meta-analysis. *PLoS ONE*, *8*(12), e85179. https://doi.org/10.1371/journal.pone.0085179

Kasturiratna, K. T. A. S., Hartanto, A., Lee, A. K., Koh, C. J., & Majeed, N. M. (2025). Efficacy of digital mental health interventions for attention-deficit hyperactivity disorder (ADHD): A meta-analytic review of randomised controlled trials. *Computers in Human Behavior Reports*, *19*, 100703. https://doi.org/10.1016/j.chbr.2025.100703

Katebi, A., HajiZadeh, M. H., Bordbar, A., & Salehi, A. M. (2022). The relationship between “job satisfaction” and “job performance”: A meta-analysis. *Global Journal of Flexible Systems Management*, *23*(1), 21–42. https://doi.org/10.1007/s40171-021-00280-y

Landis, J. R., & Koch, G. G. (1977). The measurement of observer agreement for categorical data. *Biometrics*, *33*(1), 159. https://doi.org/10.2307/2529310

Lua, V., Ooi, W., Najib, A., Tan, C., M. Majeed, N., Leung, A., & Hartanto, A. (2023). Think your way to happiness? Investigating the role of need for cognition in well-being through a three-level meta-analytic approach. *Motivation and Emotion*, *48*, 1–25. https://doi.org/10.1007/s11031-023-10047-w

Majeed, N. M., Chua, Y. J., Kothari, M., Kaur, M., Quek, F. Y. X., Ng, M. H. S., Ng, W. Q., & Hartanto, A. (2023). Anxiety disorders and executive functions: A three-level meta-analysis of reaction time and accuracy. *Psychiatry Research Communications*, *3*(1),

100100. https://doi.org/10.1016/j.psycom.2022.100100

Majeed, N. M., Hartanto, A., & Tan, J. J. X. (2021). Developmental dyslexia and creativity: A meta-analysis. *Dyslexia*, *27*(2), 187–203. https://doi.org/10.1002/dys.1677

McHugh, M. L. (2012). Interrater reliability: The kappa statistic. *Biochemia Medica*, *22*(3), 276–282.

Ogilvie, J. M., Stewart, A. L., Chan, R. C. K., & Shum, D. H. K. (2011). Neuropsychological measures of executive function and antisocial behavior: A meta-analysis. *Criminology*, *49*(4), 1063–1107. https://doi.org/10.1111/j.1745-9125.2011.00252.x

O'hara, M. W., & Swain, A. M. (1996). Rates and risk of postpartum depression—A meta-analysis. *International Review of Psychiatry*, *8*(1), 37–54. https://doi.org/10.3109/09540269609037816

Ong, A. D., Thoemmes, F., Ratner, K., Ghezzi-Kopel, K., & Reid, M. C. (2020). Positive affect and chronic pain: A preregistered systematic review and meta-analysis. *Pain*, *161*(6), 1140–1149. https://doi.org/10.1097/j.pain.0000000000001828

Willis, L. D. (2023). Formulating the research question and framing the hypothesis. *Respiratory Care*, *68*(8), 1180–1185. https://doi.org/10.4187/respcare.10975

Wong, J., Yi, P. X., Quek, F. Y. X., Lua, V. Y. Q., Majeed, N. M., & Hartanto, A. (2024). A four-level meta-analytic review of the relationship between social media and well-being: a fresh perspective in the context of COVID-19. *Current Psychology* (New Brunswick, N.J.), *43*(16), 14972–14986. https://doi.org/10.1007/s12144-022-04092-w
